# A narrative systematic review of factors affecting diabetes prevention in primary care settings

**DOI:** 10.1371/journal.pone.0177699

**Published:** 2017-05-22

**Authors:** Josie Messina, Stephen Campbell, Rebecca Morris, Emily Eyles, Caroline Sanders

**Affiliations:** 1 School of Primary Care, University of Manchester, Manchester, United Kingdom; 2 School of Geographical Sciences, University of Bristol, Bristol, United Kingdom; University of South Australia, AUSTRALIA

## Abstract

**Background:**

Type 2 diabetes is impacting millions of people globally; however, many future cases can be prevented through lifestyle changes and interventions. Primary care is an important setting for diabetes prevention, for at-risk populations, because it is a patient’s primary point of contact with the health care system and professionals can provide lifestyle counselling and support, as well as monitoring health outcomes. These are all essential elements for diabetes prevention for at-risk adults.

**Aim:**

To understand the factors related to the delivery and uptake of type 2 diabetes prevention interventions within primary care in higher income countries.

**Methods:**

For this narrative systematic review, we combined qualitative and quantitative studies of diabetes prevention within a primary care setting for patients at-risk of developing the condition. We used an iterative approach for evidence collection, which included using several databases (MEDLINE, Embase, Pysch info, BNI, SSCI, CINAHL, ASSIA), where we combined diabetes terms with primary care terms. Narrative and thematic synthesis were utilised to identify the prominent themes emerging from the data.

**Results:**

A database of 6646 records was screened by the research team, and 18 papers were included. Three major themes were identified in this review. The first theme of context and setting of diabetes progression includes the risk and progression of diabetes, primary care as a setting, and where the responsibility for change is thought to lie. This review also found mixed views on the value of preventative services within primary care. The second theme focused on the various patient factors associated with diabetes prevention such as a patient’s motivation to modify their current lifestyle, perceptions and knowledge (or lack thereof) of the impacts of diabetes, lack of follow-up in healthcare settings, and trust in healthcare professionals. The third theme was centred on professional factors impacting on diabetes prevention which included workload, time constraints, resources, self-efficacy and knowledge as well as professionals’ perception of patient motivations towards change.

**Conclusion:**

This review explored the factors influencing diabetes prevention in primary care, and identified the context of prevention, as well as patient and professional factors related to preventative services being offered in primary care. This systematic review complements previous reviews of real-world settings by exploring the significant factors in prevention, and the findings are relevant to academics, policymakers, patients and practitioners interested in understanding the factors associated with the delivery and uptake of diabetes prevention interventions.

## Introduction

Type 2 diabetes, a chronic medical condition that is increasing globally at alarming rates, is recognised as a serious international health concern [1–6]. The global prevalence of diabetes in 2013 was estimated at 382 million adults with numbers expected to escalate to 592 million people by 2035 [7]. Diabetes is currently the fifth leading cause of mortality internationally [8]. Diabetes is linked to various risk factors such as obesity, sedentary lifestyles, poor dietary intake, family history of the condition, as well as socio-economic status, and ethnicity [9–11]. Research has suggested that nearly 80% of type 2 diabetes cases can be avoided through lifestyle modifications such as diet, exercise, weight management and healthy lifestyles [12–13]. Diabetes prevention interventions have been successful in reducing the risk of progression to type 2 diabetes in patients with impaired glucose tolerance [14–18]. Impaired glucose tolerance is also known as pre-diabetes, and acting quickly is essential, as a delay in diagnosis can cause damage to the body even with mild symptoms [19–21]. A recent systematic review has shown that modest lifestyle changes can reduce diabetes incidence by over 50% in at-risk individuals, and for all diabetes prevention programs compared with regular care, the pooled incidence rate was 26% lower (95% CI 7–42%) [14].

Primary care can be a valuable setting for preventing diabetes in at-risk populations as patients can be offered support for prevention [22–23], such as screening and lifestyle advice, by primary care health professionals such as GPs, practice nurses or health care assistants. Effective prevention programs have focused on using behavioural theories to encourage physical activity and improve dietary intake, such as components of tailoring, counselling, goal-setting, providing on-going feedback, and monitoring of at-risk participants [15, 24–25]. However, an outstanding challenge is translating the success of these trials into real world clinical settings such as primary care where resources, time, and other constraints exist [26]. Little is known about how health practitioners discuss lifestyle modifications with patients in primary care. Previous reviews have not focused exclusively on primary care [27–28], and those that have a primary care emphasis have focused on effectiveness of interventions [14].

Thus, there is a need for research to understand how lifestyle risk factors are addressed within primary care [29]. Therefore, this review will address the following research question: ‘what are the factors related to the delivery and uptake of type 2 diabetes prevention interventions within primary care?’ Further, it aims to explore how patients and health professionals discuss and conceptualise diabetes prevention within a primary care setting in higher income countries.

## Methodology

### Search strategy and study selection

This narrative systematic review took an iterative approach, which allowed for revisions to the inclusion and exclusion criteria (Table 1), search strategy (S1 File), as well as the core research questions after consideration of the evidence [30]. This approach was adopted as there was limited evidence for the factors affecting diabetes prevention within primary care settings. An information specialist and disciplinary experts were consulted in the formulation of the search strategy and research questions for this project. MEDLINE (Ovid), Embase (Ovid), British Nursing Index (ProQuest), Applied Social Science Index and Abstracts (ASSAI) (ProQuest), Social Science Citation Index (Web of Science), PYSCH INFO (Ovid), and CINAHL (EBSCO) were searched up to January 2015. To create a comprehensive search strategy, the ‘building blocks’ approach, a common strategy in reviews, was utilised and search terms were divided into concepts and expanded with synonyms linked with Boolean operators [31]. In addition, the ‘Berry Picking’ approach, often used in iterative searching, allowed for the search strategy to evolve with information generated from the review process [32,33]. As new evidence was revealed through the search process, the review approach allowed for the strategy to change in light of new evidence. Also, the ‘drop a concept’ searching technique allowed for stacking of terms approach to be used by firstly combining all term/concepts of the review then removing the least relevant concepts to cast a wider search net [31]. In the end, diabetes terms (i.e. type 2 diabetes, pre-diabetes etc.), primary care terms (i.e. primary care or primary health care or primary medical care etc.), and interventions terms (i.e. prevention, diabetes prevention, lifestyle advice etc.) were combined within the databases (see S1 File for sample).

**Table 1:** Inclusion and exclusion criteria.

Inclusion criteria	Exclusion Criteria
**Populations**	
• At-risk of developing type 2 diabetes through family history, obesity, lifestyle factors • Primary care staff (clinicians, nurses, and support staff)	• Non-primary care providers • Low-risk patients, those who already have type 2 diabetes or gestational diabetes
**Reported Findings & interventions**	
• Views and experiences of diabetes prevention within primary care among patients and providers • Interventions that focused on primary care staff helping patients prevent diabetes (lifestyle interventions)	• Diabetes prevention strategies outside primary care settings such as hospitals or community centres • Interventions other than lifestyle advice (such as pharmacological interventions)
**Study Design**	
• Published qualitative, mixed methods, or quantitative studies	• Websites, blogs, anecdotal evidence
**Countries, dates, language**	
• Developed countries part of the Organisation for Economic Co-operation and Development (OECD) • 1990 onward • Studies reported in English	• Countries outside of the OECD

Databases may be poorly indexed or traditional searches may result in not casting a wide enough net on the evidence [34], so in addition to the core database searching, we searched reference lists and used citation searching of included papers, as well as key author searches (completed April 2015). One of the primary exclusion criteria was the rejection of papers about contexts outside of primary care (such as hospitals or community centres). Papers including those who already have type 2 or gestational diabetes and those of low-risk were excluded from this review. The rationale for this stems from the focus of this review on diabetes prevention in primary care settings where a gap in the literature exists.

### Data extraction and quality assessment

Records were stored in a Reference Manager database and were screened at the title and abstract level by the lead reviewer (JM), and were verified by members of the research team (CS, SC, RM) for rigour and completion. The verification process for this review included secondary blind screening of 30% of the original database of 6646 records, double checking of all included and excluded papers against the inclusion criteria, and regular meetings to discuss the review process with CS, SC, and RM. A piloted data extraction tool used in similar reviews was chosen to extract study details, participant characteristics, study findings, and quality [35]. All data extraction sheets were completed by the lead reviewer (JM) and were checked for completion and accuracy by another member of the team (CS, SC, RM). Study quality was assessed by the lead reviewer (JM) and verified by authors (CS, SC, RM). We used a modified Critical Appraisal Skills Program (CASP) checklist that was adapted to suit both qualitative and quantitative studies. We examined elements of methodological appropriateness, data collection, and analysis, as well as the clarity and validity of findings which we used to assess the strengths and weaknesses of each study rather than as criteria for review inclusion or exclusion [36]. By assessing the studies on these elements, we were able to identify study strengths and potential biases or weaknesses.

### Data analysis

**Fig 1:**
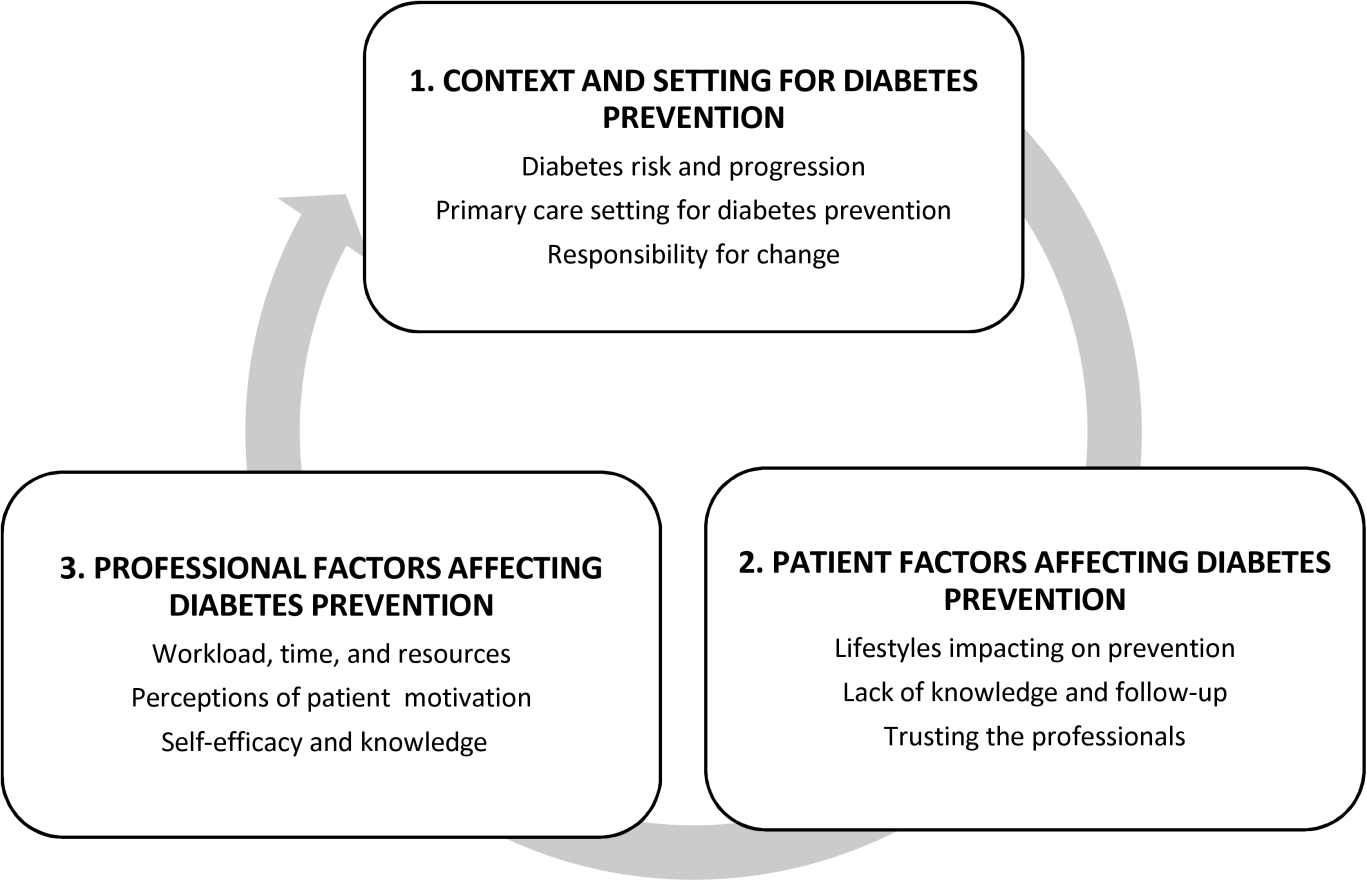
Thematic map for narrative review of diabetes prevention in primary care.

Traditional systematic reviews often focus on measures of effectiveness, provide good quality evidence-based data to answer specific questions about intervention success but often fail to provide contextual evidence [37, 38]. Thus, this narrative systematic review aimed to systematically summarise relevant quantitative and qualitative literature on patient and practitioner views and experiences of primary care diabetes prevention for patients at-risk of type 2 diabetes. Data analysis was guided by narrative synthesis and thematic analysis which identified the prominent themes emerging from the evidence [38, 39]. This method explored a much wider evidence base including investigation of views data from both qualitative and quantitative surveys [37], which allowed for the grouping of common theme categories to capture experiences, views, and social contexts of diabetes prevention in primary care within the included studies [38–40]. Once data extraction tables were completed, the lead reviewer (JM), used qualitative software (Nvivo) to organise the data from all included studies into categories which were then discussed with the review team (CS, SC, RM) to agree a coding frame which formed the three larger themes for this review (see Fig 1). Sub-themes emerged as they fit within the larger themes but were not deemed to be significant enough to be considered as a main theme. All major themes and sub-themes were verified by members of the review team with any discrepancies discussed until consensus was reached. It should also be noted that when creating themes, we took into consideration multiple papers from the same study so they did not bias the formation of a major theme but were still accounted for in the review.

## Results

### Identification of studies

**Fig 2:**
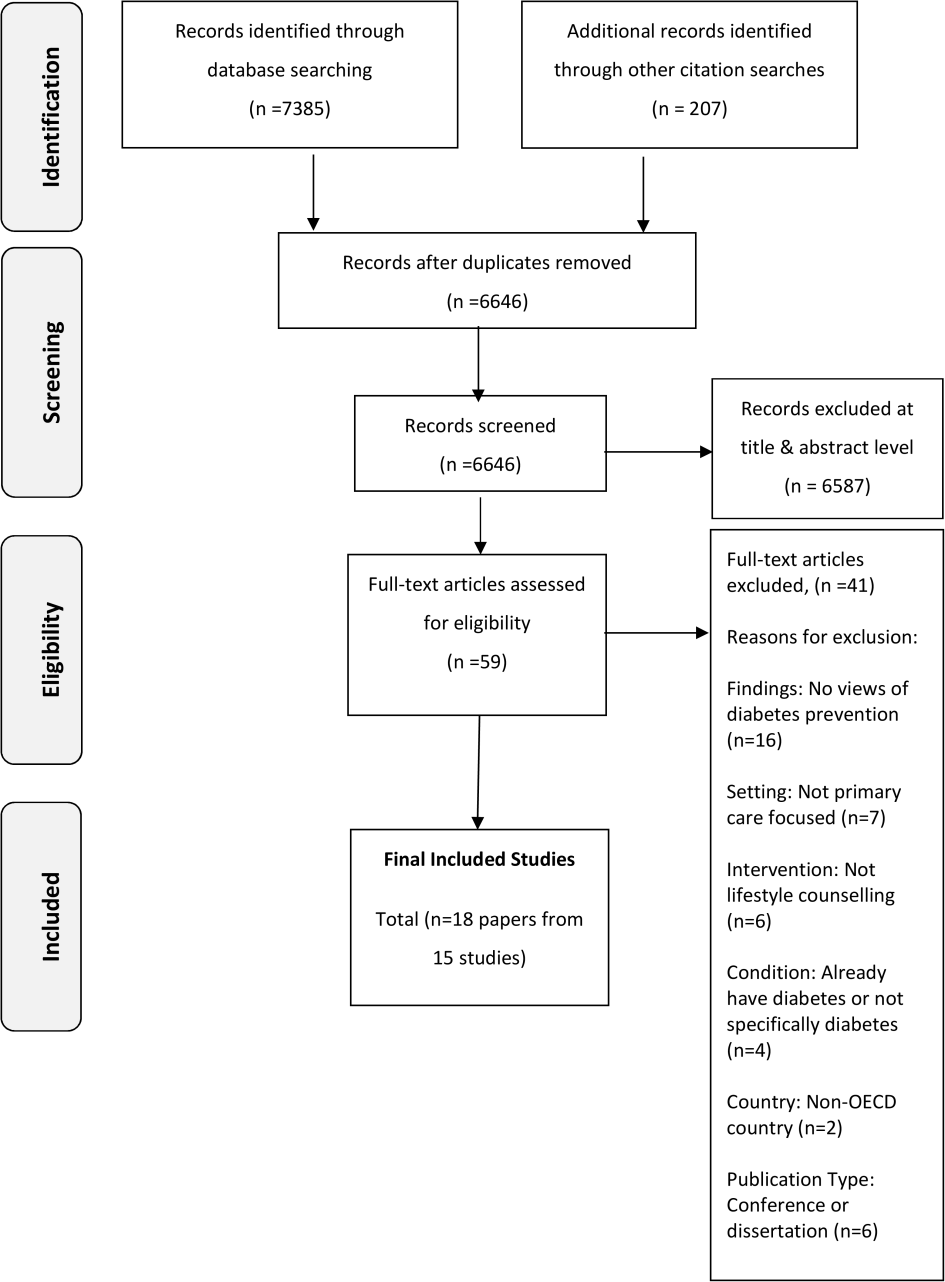
Paper selection process.

The database search resulted in a total of 6646 records (after duplicates were removed) from several databases. In keeping with the iterative search approach, additional papers were screened after undertaking citation searches, reference list searches, and key author searches. Full text papers were judged against the inclusion and exclusion criteria (Table 1), and a total of 18 papers from 15 studies were included in this review [41–58] (Fig 2). Qualitative software (Nvivo) was used by the lead reviewer (JM) to organise the extracted data from each paper into larger themes and then sub-themes which were verified by the research team.

### Summary of included studies and quality

All studies included in this review had a primary care focus and were conducted in the UK (n = 6), the Netherlands (n = 3), Finland (n = 1), USA (n = 2), Australia (n = 2), Canada (n = 1). The studies were set within a primary care setting and were mainly qualitative; however, some studies utilised mixed methods (n = 3 studies from 4 papers) [41–43, 52] and quantitative approaches (n = 2 studies from 3 papers) [44, 45, 51]. Further details on sample size and included participant characteristics are represented in (S2 File). Papers included in this review expressed the views of primary care health professionals, such as General Practitioners (GPs) or nurses, (n = 5), patient views (n = 3), and many of the papers combined professional and patient views (n = 10).

### Study quality

Many included studies were clear in their objectives and selected methods that were appropriate to address this study’s aims. Details of study quality can be found in (S2 File). Most studies recruited a self-selected sample, so their results were affected by selection bias and information bias. This may impact on internal validity, possibly impacting on the quality of quantitative studies, though to a lesser extent for qualitative studies. Also, for some observational studies, small sample sizes and methodology limited the transferability and generalisability of findings. For qualitative studies, sample sizes and generalizability were not as important as these studies provided rationale for study sampling and approach to data analysis. A few studies, however, were not as clear in the reporting of their findings. Socio-demographic data mainly reported on gender and age. Income, ethnicity, and education were poorly reported in most studies so understanding the context behind study findings was difficult. More complete socio-demographic reporting in studies would have enabled a better exploration of contextual factors of diabetes prevention such as the role of age, gender, income or ethnicity on diabetes prevention. We aimed to include studies that examined views of diabetes prevention in primary care. This meant acknowledging the above-noted pitfalls in the quality of included studies in this review as they are common in observational and qualitative studies, and more likely to be impacted by bias.

### Thematic analysis

This narrative systematic review identified three overarching themes: (1) context and setting for diabetes prevention (2) patient factors affecting diabetes prevention (3) professional factors affecting diabetes prevention. From these major themes sub-themes emerged to form a narrative of patient and provider experiences of diabetes prevention within a primary care context (Fig 1). This review features themes that emerged from the data and utilises quotes and statistics from included studies for illustrative purposes.

## Main themes

### 1. The context and setting for diabetes prevention

#### Diabetes risk and progression

A major theme identified in eight of the studies was the context of diabetes risk and progression [38, 46–50, 53]. In a survey study, all GPs were aware of pre-diabetes as a condition, however, 47% were unaware of the risks of pre-diabetes progressing to diabetes [43]. Health professionals and patients valued prevention and understood that Type 2 diabetes was a real condition with potentially severe complications if not carefully managed:

*“If I make no changes*, *if I decide to make no changes*, *I will become a diabetic*, *I will have heart disease*.*”* (Patient, [46], p.773)

Diabetes risks were linked to family history, obesity, and lifestyle factors such as diet and physical activity [46–50]. Patients based their diabetes risk on whether they personally believed they were at higher/lower risk of developing the condition, and often cited heredity as a reason for increased risk, and lifestyle and lack of family history for lower perceptions of risk [49], although a theme of uncertainty was evident due the complex risk factors [49,50]:

*“I don’t know what they found to make them think I am at risk in the future… what would make them believe that I will develop diabetes*. *I don’t know why*?*”* (Patient, [50], p.90).

Furthermore, a study within a Bangladeshi community found some participants believed genetics, stress, and social isolation were linked to diabetes progression, while some believed diabetes was widespread and common in the community and not a worry [47,48].

Health professionals explained the risks of developing diabetes and how it can be prevented:

*“She [health professional] explained… if you are careful and all things like that you can stop it from being diabetic which is…nice to know …”* (Patient, [46], p.773)

#### Primary care as a setting for diabetes prevention

A mixed commentary on primary care as a setting for diabetes prevention activities was featured in eight papers [42, 44, 45, 51–54]. Evidence suggested primary care was a familiar place for most patients [37, 40], primary care should tackle prevention [46, 54], and preventative services could fit into current practices [52]; although prevention was lacking in the health service [47, 51]. However, there was some evidence pointing to resistance of making primary care into a specialist service as it was seen as expensive, time consuming, and outside of the remit of a generalist service such as primary care [51]. Some professionals were unconvinced about the evidence supporting the effectiveness of diabetes prevention in primary care outside intensive trials [43, 51], and one survey found that 23% health professionals believed the chances of success in lifestyle interventions was low [45]:

*“The evidence for actually preventing these people going on to develop diabetes involves very intensive*, *expensive lifestyle intervention regimes*, *so the evidence that the little bit that we do is actually making an impact probably is not there*.*”* (GP, [51], p.533)

In addition, two studies found professionals believed screening and treating pre-diabetes was essentially ‘medicalising’ something that is seen by them as more of a social problem [43, 51]:

*“These people [those with impaired glucose tolerance and impaired fasting glycaemia] are not ill*. *Should we make them ill*?*”* (GP, [51], p.534)

#### Responsibility for change

Responsibility for change was a noted theme in five studies [44–46, 49, 51] from four European countries, with one from Australia, where diabetes prevention is part of routine services in primary care. As part of their role, GPs and nurses can act as supporters for lifestyle change [45] by taking on the role of facilitators and advisors to empower patients to improve their health [46]; however, the onus of change rests with the individual [49,51]. For example, in one study the “majority of physicians (88%) and nurses (95%) agreed that patients themselves must accept the responsibility for lifestyle-related decisions” [44] (p.264). It was noted that these changes to lifestyle can be difficult but support from professionals can help:

*‘I think they have an enormous responsibility to actually bear on their patients to [start] living in a healthy manner*.*’* (Patient, [49], p.330)

This theme also links to evidence presented later in this paper on professional factors affecting diabetes prevention such as workload, contexts and professional support for prevention.

### 2. Patient factors affecting diabetes prevention

#### Current lifestyle impacting on diabetes prevention

Various papers made reference to how current lifestyles acted as a barrier to change [42, 45, 47, 49, 54, 55]. For example, a Dutch study found the temptation to snack was a challenge for participants, though some participants reported little difficulties in sticking to dietary protocols [55]. The same study also found patients believed their current way of life was sufficient for preventing diabetes with many patients stating they met standards for diet and physical activity, and 26% stating they were satisfied with their health behaviour [55].

Many reasons were given to explain why lifestyle changes were not sustained. For example, being pushed for time [42, 47] made it difficult to sustain lifestyle changes such as physical activity and preparing healthy meals were reported [49]. In one feasibility study of a lifestyle program in primary care reported patients viewed maintaining a healthy diet as ‘expensive and time consuming’ [42]. Furthermore, another study reported issues of prevention programs not being offered at suitable times around working hours:

*“… if these people are working they can’t [attend programs] unless it’s delivered in the hours that are suitable*… *you’re talking about you know targeting people in their 30s*, *40s*, *50s and they’re still working*.*”* (GP, [54], p. E)

Costs associated with healthy lifestyles were also seen as a barrier especially if attending programs that were not free of charge [54]. In addition, making lifestyle changes could include incurring additional costs such a gym memberships, purchasing healthier options, or paying to attend programs:

*“Cost probably was ‘cause I used to go to a gym and I was working and it was getting expensive as well and I wasn’t getting there very often*, *so yeah I stopped going*.*”* (Patient, [49], p.330)

Other constraints on lifestyle change included: reliance on unhealthy convenience foods financial constraints, childcare issues, safety outside locality, and language barriers within health care and in the community [47]. In addition, stress, financial factors, difficulties with maintaining dietary restrictions, weather, and disability and pain were noted as barriers for engaging in changes [42].

#### Lack of knowledge

Three studies reported on lack of knowledge as impacting on diabetes prevention in primary care [44,46,49]. A study which aimed to develop a diabetes prevention pilot program found both patients and practitioners lacked knowledge on pre-diabetes [46]. Patients could benefit from written information to help them understand pre-diabetes risks and causes [46]. In another study, respondents who were diagnosed with pre-diabetes were provided with written information. However, most participants in this study thought it was too vague and unhelpful [50]. There was often a ‘grey area’ for pre-diabetes’:

*“I am borderline diabetic*, *I’m in that grey area*, *not quite diabetic but I could be*” (Patient, [50], p90)

Also, a lack of knowledge of diabetes made it difficult for participants to understand the condition, the symptoms, how it affects patients, and how to prevent it from developing [50]. Most patients were keen to prevent diabetes but struggled to understand how to take preventative action [50]:

*“I want to prevent it if I can*, *and I don’t know how*. *I am up in the air and hoping”* (Patient, [50], p.91)

The evidence above points to the importance of knowledge about diabetes in its prevention. In a survey study seeking professional views on prevention, the majority of health professionals felt information provision was part of their role in supporting patients [44]. In this study, the GPs and nurses felt insufficient patient knowledge was rarely mentioned by patients as a barrier for making changes [44].

#### Lack of follow up

Four studies reported lack of follow up in primary care diabetes prevention [42,46,49,54].

“*You*’*ve got me so far down the path and left me*, *and I have a number of paths to choose now*. *I don*’*t know which one to choose and I might just walk back*. *It is not satisfactory to screen people and not follow them”* (Patient, [50], p.91)

Health professional respondents who offered a preventative diabetes intervention believed continuity of care was crucial and their patients would not sustain changes without long-term follow up [54]. This view was also held by patients suggesting it would be difficult to maintain change without on-going professional follow up [42]. Patients in one study indicated they would have valued a visit with a professional after diagnosis, and all patients wanted repeat pre-diabetes tests as a means of monitoring and feedback [50]. Lack of follow up was an issue for patients and providers in a pilot study suggesting regular follow up and a pre-diabetes registers could be a way to address the issue [46].

#### Motivation through program involvement

Six studies examined patient motivators for change in the areas of involvement in programs and how these programs changed their perspectives on health [41, 42, 46, 49, 54, 58]. Some patients were motivated to make changes by simply taking part in a group program: *“I need a group to get me going”* [41] (p.15), formal meetings and completing programs were motivators for some [42], as well as on-going follow- up and behavioural monitoring [42, 54]. Taking part in a program could also be an opportunity to learn more about diabetes risk factors such as diet and exercise [41].

*“I found that people who are ready to change took the info and did the interventions [and] were motivated*. *Others did not*, *but most made some small change*, *which is positive*, *anything is better than nothing”* (Nurse, [54], p. E)

The atmosphere and context in which the advice was provided was also important in one study with an informal and relaxed atmosphere was seen as a positive feature [41]. Respondents from a feasibility study valued realistic goal-setting and having a peer group support [54]. Providing advice that mirrored a patient’s concerns, provided relevant goals, and used concrete examples could also help build motivation towards change: “*You just try to hook onto something that is important to them”* (Professional, [58], p.462). In a pilot program, patients made use of study materials, and most participants were able understand diabetes and how to take actions forward to prevent diabetes, suggesting the value of tailored relevant programs to patients [46].

#### Trusting the professionals

Five studies examined how trust and confidence in health professionals impacted on diabetes prevention efforts [42, 46–50]. Some patients relied on their professional to help them gauge the seriousness of the problem in two studies [46,50], and a consultation with a professional and scheduled follow-ups were interpreted by a patient to be a marker of a serious issue. A familiar face in primary care had a positive impact on prevention [42], and provider attributes such as professionalism, honesty, and trust helped patients make informed decisions about their health:

*‘But I tend to follow*, *the advice of the doctor it is for a good reason and it’s*, *and I would be stupid if I didn’t take the advice of the doctor*.*’* (Patient, [49], p.330).

### 3. Professional factors affecting diabetes prevention

#### Workload, time, and resources

The most noted factor related to diabetes prevention in primary care was workload and resources which was expressed in eleven papers [43–46, 51–54, 56–58]. Papers reported professional worries of being swamped and unable to manage the additional workload [43, 46, 51, 56], lack of time [44, 45, 54, 56–58], disturbed workflow [57], resources already being stretched [43, 51], and financial and organisational concerns [43, 45, 51, 53, 54, 57]:

*“… if we do find diabetics then we’re going to have to manage them*, *so I think what would be the implication is time and that could well be a barrier”*. (Nurse, [56], p.157).

Another important factor in diabetes prevention was competing health priorities and dealing with pre-diabetes alongside other health conditions [43, 54]. This issue of managing health priorities in a consultation with a patient was noted as an obstacle for identifying and addressing pre-diabetes in primary care:

*“Fine*, *yes*, *in theory [we could screen for impaired glucose tolerance]*, *but we haven't only even got diabetes to look after*… *but you've got so many things to look after and outside issues as well*, *so where does it stop*?*”* (GP, [43], p.4)

While the papers examined in this review featured workload and resources as a barrier to diabetes prevention service in primary care, screening, diagnosing, and following up with patients was also seen an essential part to diabetes services in one paper [46]. For example, GPs and nurses felt providing diabetes prevention was part of their role in primary care [44], and prevention services were already part of professional’s workload [52, 54], as it was easy to incorporate prevention as part of health checks and routine care [51,54].

*“You should be seeing them anyhow early because they are hypertensive or become hypertensive and that is how they get picked up as being impaired glucose tolerance*, *so they are already a population that we are probably seeing”* (GP, [51], p.533)

The evidence presented in the above paragraph was from mainly European countries and Australia, where diabetes prevention within primary care is driven by a policy mandate that may have had a knock-on effect on practice staff’s attitude towards their role in preventing diabetes in their patients.

Training and practice support for diabetes prevention was noted as helpful in providing a more informed and systematic approach [54], and viewed by nurses to be valuable [45], although some nurses got better at delivering the program over time and were successful with little training [57]. In a survey study examining professional motivation, 73% stated they felt supported by practice staff [52], and practices nurses were vital in the sustainability of diabetes prevention efforts [54].

#### Perception of patient motivation impacting on professionals’ advice giving

Professionals’ perception of patients’ motivation to change was noted as a factor impacting on diabetes prevention in primary care in eight studies [42–45, 47, 48, 51, 58]. A health professional could be deterred from providing lifestyle advice to those perceived to be unwilling patients [44], older patients who were perceived as unlikely to make changes [56], or asymptomatic patients [43, 51]:

*“… we have diabetics who*… *who just totally ignore the advice you give them*, *and I think going further back than that and giving them advice when they haven't got diabetes as such is going to be very difficult*” (Professional, [43], p.4)

A study within the Bangladesh community found health professionals had a poor cultural understanding of Bangladeshi patients and perceived them as reluctant to engage with lifestyle change [47, 48]. However, professionals would like more training in cultural awareness. Further, an implementation study found nurses struggled with motivational interviewing as it was difficult to motivate patients to change; however, nurses felt prepared and effective when delivering the program in an informal and unstructured manner [42, 57].

#### Self-efficacy and knowledge

Another factor relating to diabetes prevention was professional knowledge and self-efficacy which was reported in five papers [44–46, 52, 58]. A lack of knowledge about pre-diabetes was noted in one qualitative study [46], and also a lack of specialist dietary knowledge was mentioned by professionals as a barrier for guiding discussions on behaviour change [45]. In one study while health professionals believed that providing lifestyle change advice was part of their role, only slightly more than half of these respondents believed their skills in this area were adequate [44]. Low self-efficacy in clinical abilities relating to identifying diabetes risk was a barrier in a qualitative study: “I *have very little confidence in my ability to quantify somebody's risk”* (Professional, [58], p.462). However, on the contrary, a quantitative study found professionals were confident in their skills and advice giving [45]. Similarly, a mixed method study suggested over one third of professionals reported high self-efficacy [52].

## Discussion and conclusion

### Summary of findings

This review has examined the factors relating to diabetes prevention in primary care by systematically reviewing the published literature in this area (Summarised in Table 2). We found diabetes prevention to be impacted by a patient’s desire to modify their current lifestyle, and perceptions and knowledge of the impacts of diabetes. Patients were also influenced by contact with a trusted professional who can help motivate them towards making healthier lifestyle changes that could ultimately lead to a reduction in diabetes risks. Difficulty in changing current lifestyle, lack of time, costs, and personal difficulties were noted as obstacles for prevention for patients. This review also points to professional factors such as workload, knowledge of the professional, willingness to provide advice, and confidence in abilities to provide diabetes prevention advice as playing a big role in preventative services.

**Table 2:** Summary of review findings and implications for practice.

Main theme	Supporting sub-themes from analysis	Implications for practice and research
Context and setting for diabetes prevention	GPs and patients aware of diabetes but more knowledge required to fully support prevention	Training of health professionals and education of patients to gain a better understanding of how diabetes risks can be reduced through lifestyle change
	Family history and lifestyle risk factors	
	Mixed views about primary care as a setting for prevention	Further qualitative research with health professionals exploring primary care as a setting for diabetes prevention
	Role of health professional as supporters in assisting patients with lifestyle change	Training and support offered to health professionals to enable them to effectively support their patients in making changes. This could include education days, brief computer prompts to discuss lifestyle with patients, a plan for patient follow up.
Patient factors affecting diabetes prevention	Difficulties with engaging and maintaining lifestyle change as well as practical constraints as a barrier	Lifestyle change is a difficult process for patients, so primary care professional can help support the patient along the way. Support could include repeat blood tests, increased follow up, referrals to community programs (activity or weight loss for example)
	Consultation, monitoring, and follow up with a trusted professional essential in preventative services	Primary care centres can create a diabetes prevention program which could include plans for screening, lifestyle advice, monitoring and feedback
Professional factors affecting diabetes prevention	Workload, competing interests, and resources impact on the delivery of services, although prevention can fit into existing workloads	Future research could explore these factors more in-depth Primary care centres can prioritise prevention and set up systems to encourage staff to screen, monitor, and follow up patients
	Professionals can be deterred from providing preventative services due to perceived lack of patient motivation	Personal and tailored plans can be created with the patient to ensure realistic and achievable goals are set
	Mixed views of professionals held knowledge and self-efficacy in delivering prevention	Training and education for health professionals could increase their knowledge and self-efficacy

### Relationship with existing literature

Pre-diabetes, a pre-cursor for diabetes identified through blood tests, is a condition that is asymptomatic [20]. Receiving professional help and screening for this condition may not occur even though patients are at-risk [59]. As patients may not feel ill, they may be unlikely to take serious actions to prevent consequences that may be less immediate [22]. However, if patients were identified prior to developing full diabetes, nearly 80% of type 2 diabetes cases could be avoided through lifestyle modifications [12], which represents an opportunity for primary disease prevention rather than the treatment of patients who already have the disease. A recent systematic review examining translational real-world studies, such as healthcare or community settings, found support that lifestyle change programs can also significantly reduce diabetes progression [14].

In addition, findings from this review point to the importance of patient and provider knowledge of the seriousness of diabetes and understanding of diabetes risks factors such as weight and family history; however, our review also highlighted the lack of knowledge about diabetes among both patients and providers. This could have implications for diabetes prevention as a patient will assess their lifestyle, risk factors and genetics to determine health risks [60], and personal conceptualisation of illness can have a great impact on how a patient will manage their risk factors for a condition [61], with patients less accepting of their high risk status being those less inclined to change [62]. Similarly, Heisler et al. [63] found patients have an unmet need for information, with many patients leaving their consultations with insufficient diabetes knowledge. Nam and colleagues [64] argued that unsatisfactory medical outcomes in diabetes, such as glucose control, reflected a lack of patient knowledge, but also a failure of the professional to provide appropriate interventions. To combat lack of knowledge of pre-diabetes, professionals should focus on educational dialogues and personalised care plans developed by patients and providers [65]to avoid issues of lack of uptake and maintenance of lifestyle change [64].

Our findings also found mixed views on the primary care setting for diabetes prevention, with some evidence in support of primary care services, and some suggesting providers are unconvinced about preventative services in primary care [14]. However, some suggested programs offered in primary care may require more evidence [66]. A major concern for such programs would be replicating the results of successful interventions [14, 16, 67, 68] with limited resources which is an important area of research and policy [58, 66, 69, 70]. Diabetes prevention programs have a focus on lifestyle improvements which are “notoriously difficult to achieve and sustain,” especially those involving weight loss and exercise [70] (p.7).

Another theme presented in this review was professional factors impacting on care such as workload, resources, self-efficacy, and the role of perceived patient motivation acting as a barrier or facilitator for lifestyle advice. While the patients are ultimately responsible for lifestyle changes, physicians also carried the burden of administering advice and setting goals with patients, but workload concerns and lack of time were considerable factors [59]. Primary care, which is commonly the venue for diabetes services, is often under strain with professionals under increasing pressure to address competing, multiple health care problems at any given health care visit [66]. Some of these challenges may result in health practitioners abandoning health lifestyle change interventions since they may feel unprepared or worried about threatening patient rapport [71]. Prioritising diabetes in primary care could be strengthened by focusing on staff training and educational initiatives to foster health professional self-efficacy as well as streamlining preventative services to maximise efficiency to help combat workload issues.

Our review has also pointed to the importance of maintaining good, trusting and supportive relationships between patients and professionals so that diabetes prevention can be facilitated within primary care. Research suggested changing the way patients and providers communicated with one another could have positive impacts on improving diabetes outcomes as well as preventing the onset of diabetes in at-risk patients [58, 63, 64, 68, 72]. Patient-centred care is known to foster trust and lead to open and relaxed relationships between patients and providers [73]. Essential elements of effective patient and provider relationships should feature the ideas, feelings, and values of both parties, including open-dialogues that feature both perspectives while solving problems and creating care plans. While the evidence is strong in support of shared decision-making, work by Heisler et al [74] reported that physician dominant participatory decision-making was the paradigm most followed, but by following a more patient-centred approach, a substantial improvement was found in the communication and provision of information, something primary care diabetes programs were sometimes found to lack, particularly in respect to follow-up [46, 50].

Our review has highlighted patient motivation and desire to change in diabetes prevention efforts. Motivated patients tend to have skills, knowledge and confidence to facilitate healthy decision-making [75]. Previous research points to patients being encouraged by short-term gains rather than by the long term negative complications of diabetes [73], and patients favoured interventions that maximised benefits with minimal disruption to daily routines [76]. Motivations can impact on actions to maintain glucose control, as patients may become discouraged due to lack of results or progress, but same patients may genuinely be uninterested in altering their lifestyle since they have not experienced ill health [59]. Thus, it is important that professionals seek a balance between a patient’s desire for information and the desire for involvement, which can help reduce uncertainty [50,76]. This is important as our review has identified patient lack of knowledge and motivation as patient perceived barriers to prevention. Lifestyle interventions are often complex and can be difficult for patients to integrate into their daily lives [59, 72], thus patients and professionals need to carefully co-construct care plans [72]. While preventative efforts should be co-constructed, the majority of responsibilities for managing diabetes were imposed on patients [73, 77], and this finding is consistent with the evidence in this review.

### Strengths, limitations, and future research

Previous reviews have focussed on diabetes prevention programs [27, 28, 78], but have not specifically examined experiences within a primary care context. Thus, this review has provided a clearer understanding of the factors impacting on the delivery and uptake of preventative services to fill an evidence gap. This review has implications for how primary care practitioners deliver diabetes prevention in routine consultations, and implications for guidance for the delivery of preventative care in primary care. This review points to the importance of balancing workload and other competing priorities in primary care to allow health professionals to prioritise diabetes prevention which would include screening, education, and support and follow up; however, our results revealed mixed professional views of the value of primary care diabetes efforts. Future work could focus on seeking out views and experiences of providers and patients who do not value/offer preventative services in primary care despite compelling evidence of effectiveness and many national guidance schemes supporting prevention in primary care [14]. Findings also suggested patients lack sufficient knowledge of diabetes risk, thus initiatives to educate patients could help to promote an understanding of diabetes risk.

This review was limited by quality of evidence since all studies included a self-selected sample the findings are not easily generalisable since they may be biased towards a certain viewpoint [79]. Secondly, while this review sought to explore views and experience of diabetes prevention, we were limited by the depth and breadth of existing studies that focused on prevention in primary care, with many studies lacking depth in their findings. For example, survey studies did not offer as much detail on the views and experiences of diabetes prevention as qualitative studies. Furthermore, we lack information on the varying types of prevention initiatives within primary care settings. For example, this review could not adequately compare specific interventions between studies, as information was poorly reported in included studies. There also may be policy differences between high-income country settings, especially in primary care, given that, for example, some places may have publicly-funded universal care whereas others may not. This debate within the research regarding the role of primary care diabetes prevention is important, since for primary care to adopt this role more research is needed to understand what is enabling or constraining diabetes prevention in everyday practice, though some of these factors have been highlighted in this review. Finally, our review, while systematic, only examined published literature meaning there may be useful and relevant unpublished studies that would have been missed in a published literature searching. Future work could explore grey literature to see if they can build on the findings from this review.

## Conclusion

This review has identified various factors relevant to diabetes prevention in primary care such as the importance of understanding diabetes risks by both providers and patients, as well as how patient motivations not only drive change but also have an impact on professionals offering advice. Both patients and professionals are not always aware of the risks of pre-diabetes. With patients, care must be taken that information is helpful, as it was found that some information provided was seen by some as vague, and pre-diabetes could be seen as a ‘grey area’. Professionals working in primary care are faced with a heavy workload and resource issues, though primary care is seen as a setting where diabetes prevention should and could fit. Systems to encourage staff to screen and monitor patients with a strong emphasis on follow-up could allay both the lack of follow-up observed and the workload/resources issue. While some professionals were unconvinced with respect to the effectiveness of preventative care in the primary care setting, again, a more effective provision of knowledge could mitigate this issue. The motivation and current lifestyle of patients are barriers to change and often impact the sustainability of lifestyle changes. Also, ensuring that patients have a voice in lifestyle changes may aid in tailoring plans with realistic, sustainable, and achievable goals, as well as helping with motivation. Further research is particularly needed on the setting of prevention, and the optimal provision of knowledge to both patients and professionals. This review adds to existing literature on diabetes prevention in primary care by systematically identifying the factors related to offering diabetes prevention services in primary care settings.

## Supporting information

S1 PRISMA Checklist PLOS ONE PRISMA checklist. (DOC)

S1 File Sample MEDLINEsearch strategy. (PDF)

S2 File Extraction tables. (PDF)
