# Supplementary material for: A narrative systematic review of factors affecting diabetes prevention in primary care settings
Source: PLoS One. 2017 May 22;12(5):e0177699. doi: 10.1371/journal.pone.0177699 (PMC5439678; doi:10.1371/journal.pone.0177699)
Supplement: S1 File — (PDF) [file pone.0177699.s002.pdf]

Supporting Information

S1 Appendix

Sample Medline Search Strategy

1. Primary Health Care/

2. Diabetes Mellitus, Type 2/

3. Prediabetic State/

4. Preventive Medicine/

5. (primary care or primary health care or primary medical care or medical care or family practice* or family doctor* or family medicine or general practice* or physician* or doctor* or nurs* or health professional* or GP or general practitioner* or practitioner* or prevent* medicine).mp. [mp=title, abstract, original title, name of substance word, subject heading word, keyword heading word, protocol supplementary concept, rare disease supplementary concept, unique identifier]

6. (pre-diabet* or prediabet* or High blood sugar or abnormal blood glucose level* or dysglycaemia or dysglycemia or intermediate hyperglycemia or intermediate hyperglycaemia or insulin resistance or metabolic syndrome or hyperinsulinaemia or hyperinsulinemia or non diabetic hyperglycemia or hyperglycaemia or impaired fasting glucose or IGT or impaired glucose intolerance or impaired glucose level* or impaired glucose metabolism or impaired glucose regulation or raised glucose).mp. [mp=title, abstract, original title, name of substance word, subject heading word, keyword heading word, protocol supplementary concept, rare disease supplementary concept, unique identifier]

7. (lifestyle intervention or lifestyle advice or lifestyle educat* or lifestyle discuss* or lifestyle modif* or lifestyle chang* or lifestyle counsel* or lifestyle factor* or behav* intervention or behav* advice or behav* educat* or behav* discuss* or behav* modif* or behav* chang* or behav* counsel* or diet* or nutrition* or physical activity or exercis* or weight loss or weight management or obesity).mp. [mp=title, abstract, original title, name of substance word, subject heading word, keyword heading word, protocol supplementary concept, rare disease supplementary concept, unique identifier]

8. (prevent* or avoid* or decreas* or reduc*).mp. [mp=title, abstract, original title, name of substance word, subject heading word, keyword heading word, protocol supplementary concept, rare disease supplementary concept, unique identifier]

9. 2 and 8

10. 2 and 4

11. 1 or 5

12. 3 or 6

13. 9 or 10 or 12

14. 7 and 11 and 13
