# Supplementary material for: A narrative systematic review of factors affecting diabetes prevention in primary care settings
Source: PLoS One. 2017 May 22;12(5):e0177699. doi: 10.1371/journal.pone.0177699 (PMC5439678; doi:10.1371/journal.pone.0177699)
Supplement: S2 File — (PDF) [file pone.0177699.s003.pdf]

S2: Extraction Tables

Study details	Population and setting	Methods & Study Quality	Findings
**Author**: Evans<br><br>**Year:** 2007<br><br>**Setting / country**: UK (England)<br><br>**Aim of study:** To identify key messages about pre-diabetes and to design, develop and pilot an educational toolkit to address the information needs of patients and health professionals<br><br>**Study design**: qualitative (Focus group, expert panel, video observation)<br><br>Part of WAKE UP STUDY Development of an educational 'toolkit' for health professionals and their patients with prediabetes: The WAKEUP study (Ways of Addressing Knowledge Education and Understanding in Pre-diabetes)<br><br>**Funding**: Diabetes UK	**Number of participants**:<br><br>The initial expert reference group discussion involved seven participants.<br><br>This was followed by a total of eight focus groups involving 19 health professionals and 10 service users, as well as 11 videotaped individual consultations (involving 11 patients)<br><br>**Age Range**:<br><br>- Health professional focus groups 30-59 yrs<br>- Service user focus group 48-79 yrs<br>- Patient interviews 49-77 years<br><br>**Gender**:<br><br>- Health professional focus groups 8 were female<br>- Service user focus group 4 were female<br>- Patient interviews 6 were female	**Data collection methods**:<br><br>**Needs Assessment Phase:** Mixed qualitative methodology within an action research framework. Focus group interviews with patients and health professionals and discussion with an expert reference group aimed to identify the important messages and produce a draft toolkit.<br><br>**Intervention Development Phase**: Two action research cycles were then conducted in two general practices, during which the draft toolkit was used and video-taped consultations and follow-up patient interviews provided further data.<br><br>**Data Analysis**: Framework analysis techniques were used to examine the data and to elicit action points for improving the toolkit.<br><br>**Study Quality:**<br><br>Objectives and methods appropriate.<br><br>Data collection and analysis described.<br><br>Findings appropriately discussed with a rich account of patient experiences.	Data derived from the Needs Assessment phase data and refined throughout the research cycles found that:<br><br>• Prediabetes is a serious condition, with a high risk of progressing to Type 2 diabetes and heart disease.<br>• The good news is that these risks are often preventable.<br>• To prevent progression, patients need to make lifestyle changes in terms of healthier eating (losing weight) and increased physical activity.<br><br>**Main Themes relevant to research question**:<br><br>Key to quotations: P, patient; HP, health professional; SU, service user from reference group; FG, focus group; PCI, post consultation interview.<br><br>**Knowledge and information needs:**<br>Both patients and practitioners felt that they **lacked knowledge about prediabetes** and were keen for written materials, including information on the nature of prediabetes, its causes and risks. There was considerable variation in the level of depth and breadth of information needed.<br><br>For GPs a **laminated single page** 'clinical guideline' summary was viewed as a useful resource.<br><br>The link between **high blood sugar and risk of coronary heart** disease was not being made. In terms of dietary advice, patients wanted specific information about what **foods they should eat** or avoid, and how to manage their diets.<br><br>**Conveying knowledge and motivating change:**<br>Strong themes were evident in the health professionals' data around how health professionals could **best communicate the risks, motivate patients and support lifestyle change.** Suggestions on supporting lifestyle change included the use of group sessions and involving family members or spouses. Some patients were being enthusiastic and others feeling they would be reluctant to attend group sessions.<br><br>In the feedback on the first draft of the toolkit a number of patients felt that the **risk/fear messages needed to be conveyed more strongly**. The inclusion of these positive messages was also appreciated.<br><br>For some patients, being given detailed information and a follow-up appointment was seen as a sign that the GP or nurse considered this to **be a serious issue**, which in itself was viewed as motivational.<br><br>'I don't know if everybody would think it would be too scary, too explicitly say what happens if I don't change "[…] you don't explicitly say "if I make no changes, if I

S2: Extraction Tables

Study details	Population and setting	Methods & Study Quality	Findings
			decide to make no changes, I will become a diabetic, I will have heart disease"'. (SU959 FG Intervention Development)<br><br>'If you just give fear messages it doesn't work […] But if you also give them the skills and tools to become confident in changing, that seems to be the critical factor.' (HP908 FG Needs Assessment)<br><br>'She explained that you know, like being prediabetic and if you are careful and all things like that you can stop it from being diabetic which is you know nice to know …' (P966 PCI Intervention Development)<br><br>**The role of health professionals:**<br>Anxiety was expressed by health professionals about the fear of **being swamped with patients with prediabetes** when they already found it difficult to manage the increasing workload from patients with established diabetes. There was a consensus (after discussion) amongst health professionals that ultimately the **responsibility for lifestyle change lay with the patient.** They saw their role as one of empowerment and facilitation, acting as educational advisors and supporters. **Patients also saw the onus of responsibility as being on themselves**, and commented positively on the help and support they had received.<br><br>**Practice systems:**<br>Delivering key messages at the time of diagnosis (using the WAKEUP materials), giving lifestyle advice, monitoring of lifestyle changes and possible progression to diabetes, and monitoring and treatment of other cardiovascular risk factors were considered to be **essential components of practice systems** for managing prediabetes.<br><br>There was substantial concern among both patients and health professionals over the **current lack of follow-up** and support for patients with prediabetes. Suggestions to address this included regular follow-up (especially during the first few months after diagnosis) and the need to construct a register of patients with prediabetes.<br><br>Theme of which health professional (nurse or GP) should **carry out the initial consultation** and whether a team approach would help to reinforce the key messages. Practitioners suggested offering **a brief training course**, rather than just an information pack which would 'migrate quickly to the shelf'.<br><br>How to **minimize workload** and use external resources were also generated, including involving family members, signposting to local group activities or weight loss support groups, use of a group education approach, use of exercise

S2: Extraction Tables

Study details	Population and setting	Methods & Study Quality	Findings
			prescriptions and involving Primary Care Trust-based health promotion support services (e.g. Walk and Talk schemes, health trainers).<br><br>'There ought to be some training on it and um a bit more input I think.' (HP914 FG Intervention Development)<br><br>**Changes to practice:**<br>Towards the end of the study the health professionals from both practices reported being **much clearer** about the nature of prediabetes and the associated risks, and placed more importance on acting systematically as a team to address the problem. They also reported that they had **evolved clearer strategies** for managing prediabetes within the pragmatic constraints of their everyday workload.<br><br>'It's about clinicians making a clear recognition that this fasting glucose between 6.1 and 6.9 isn't something you hit, file and leave.' (HP906 FG Needs Assessment)<br><br>**Overall perceptions and usage of the WAKEUP toolkit:**<br>The toolkit was **positively received by both health professionals and patients**. All the health professionals used the toolkit to help explain prediabetes to patients, and a week after the consultation most of the patients said they had read the WAKEUP patient information booklet.<br><br>The written information was seen to **confirm and support the practitioner's advice**, and to be a resource to refer to. When patients were interviewed following their consultation, most were able to **provide a reasonable account of their situation**, which was consistent with the key messages, and to describe the actions they needed to take.

S2: Extraction Tables

Study details	Population and setting	Methods & Study Quality	Findings
**Author**:<br><br>Grace et al. 2008<br><br>and Grace 2009<br><br>**Year:** 2008/2009<br><br>**Setting / country**: UK, London, Tower Hamlets<br><br>**Aim of study:** To understand lay beliefs and attitudes, religious teachings, and professional perceptions in relation to diabetes prevention in the Bangladeshi community<br><br>**Study design**: Qualitative (Focus groups and interviews)<br><br>**Funding**: Diabetes UK grant number BDA:RD04/0002780	**Number of participants**:<br><br>17 focus groups in three phases:<br><br>**Phase 1**: Lay people of Bangladeshi origin n=80<br><br>37 men, 43 women who participated in 10 groups<br><br>Generation Bangladeshi: 1st (n=62) 2nd (n=18)<br><br>Mean Age: 35 (SD 2)<br><br>History of diabetes in first degree relative: Yes (n=30)<br><br>Mean (SD) body mass index: 26.4 (3.7)<br><br>Mean deprivation index: 51.5 (10.1)<br><br>**Phase 2**: Islamic scholars and religious leaders of Bangladeshi origin n=29<br><br>14 men and 15 women who participated in four groups<br><br>Generation Bangladeshi: 1st (n=25) 2nd (n=4)<br><br>Mean Age: 35 (SD 8)<br><br>BMI and deprivation not collected<br><br>History of diabetes in first degree relative: Yes (n=15)<br><br>BMI and deprivation not collected	**Data collection methods**: Qualitative study (focus groups and semi structured interviews). 17 focus groups were run using purposive sampling in three sequential phases.<br><br>**Data Analysis**: Thematic analysis, use of fictional vignettes, and PEN-3 multilevel theoretical framework was used to inform data analysis and synthesis.<br><br>PEN framework focuses on key impacts and factors on health behaviour and the intervention (used or Grace et al 2008).<br><br>**Study Quality:**<br><br>Two papers from one study.<br><br>Aims and objectives clearly stated with appropriate methods to address aims.<br><br>Analysis and findings appropriate with good account of participant experiences/<br><br>Good demographic information in 2008, 2009 lacking but cross referenced in 2008.	**Main Themes relevant to research question**:<br><br>**<u>Findings from Grace et al. 2008:</u>**<br><br>**Knowledge of diabetes** was generally high and had been gleaned primarily through experience of diabetes in a relative or friend. Most participants recognised the central role of personal lifestyle choices including diet (especially sugar and fat), excess body weight, and physical inactivity in the **development of diabetes**. They saw the condition as at least partially preventable through lifestyle change. Other perceived causes of diabetes included heredity and stress, which were seen as linked to social isolation.<br><br>A minority of lay participants thought a **family history** meant diabetes was inevitable, but most thought that **risk could be modified** through lifestyle change. Some saw the onset of diabetes as unpredictable and its impact as cataclysmic. Others saw diabetes as **widespread in their community** and (implicitly) something not to be too concerned about.<br><br>**Health professionals believed** (incorrectly) that Bangladeshis associate obesity with health and fertility, and hence significantly underestimated the willingness of this community to control weight.<br><br>Lay participants saw **physical activity** as important for mental wellbeing and a way of caring for the body, a central feature of the Muslim way of life.<br><br>**Responsibility for diabetes prevention**<br>Some lay participants believed that fear of the devastating impact of diabetes would **motivate preventive action** across the Bangladeshi community. Others, including Islamic scholars, framed prevention in a more positive but less dramatic way as part of a healthy lifestyle that all Bangladeshis should follow.<br><br>People with diabetes were labelled as **"out of control**," whereas those without diabetes were perceived as "in control" of food and activity choices, usually equated with having a routine or timetable.<br><br>**Faith** was seen as linked to individuals' confidence and motivation to change behaviour. Religious leaders were seen as **trusted sources of information** and support. They were **enthusiastic about working in partnership with health professionals** for mutual education and with a view to developing initiatives within the community for diabetes prevention.<br><br>Many **health professionals were reluctant to discuss lifestyle change in clinical**

S2: Extraction Tables

Study details	Population and setting	Methods & Study Quality	Findings
	**Phase 3**: Health professionals n=28<br><br>19 women and 1 man who participated in three groups, two men and six women in eight individual interviews<br><br>Mean Age: 41 (SD 8)<br><br>Ethnicity:<br>Bangladeshi n=7<br>Asian Other n=1<br>White n=17<br>Black n=3		**consultations**, partly because of their own poor cultural and religious understanding and because they perceived Bangladeshis as fatalistic (especially in relation to "the will of Allah") and hence resistant to education on diabetes prevention.<br><br>**Norms** potentially conflicted with efforts to achieve health related lifestyle change. In some focus groups, women felt strong pressure to conform to traditional norms and expectations; in others (with younger and second generation women), there was support for resisting them.<br><br>**Exercise** in the Western sense (designated activities with special clothing, undertaken in special places such as gymnasiums) was seen as alien to the culture and identity of many first (and some second) generation Bangladeshis. Sporting exercise for women and older people was seen as inappropriate and liable to meet with the social sanction of gossip and laughter, though some thought this pressure should be ignored.<br><br>Many Bangladeshis cited **structural constraints** to increasing their physical activity levels, including lack of time or money or inability to find childcare. The reluctance to travel beyond the immediate locality owing to fears about safety or difficulties with language created access problems for some first generation participants.<br><br>**Practical constraints** also affected dietary choices. Both male and female second generation participants reported heavy reliance on fast foods, which they saw as convenient and affordable. Traditional Bangladeshi fruits and vegetables were perceived to be expensive so were not consumed much, but first generation participants were often unfamiliar with cheaper, more readily available Western alternatives.<br><br>Lay participants identified **poor fluency in English**, especially in the first generation, as a **major barrier to accessing and understanding basic health information**. One of the consequences was a **reliance on other people**, often family members, to access and interpret health information on their behalf. Poor English also limited people's willingness to travel beyond the immediate neighbourhood (owing to difficulties in reading road names or asking directions). This resulted in total reliance on local food and exercise provision.<br><br><u>**Additional findings reported in Grace et al 2009:**</u><br><br>**Lay people of Bangladeshi origin** – even first generation immigrants who spoke no English and had little formal education – showed **good knowledge about healthy lifestyles**, and believed that following such lifestyles would help to prevent diabetes.

S2: Extraction Tables

Study details	Population and setting	Methods & Study Quality	Findings
			The link between **obesity and diabetes was well understood**, and participants were keen, in principle, to stay slim.<br><br>**Healthcare professionals** revealed a wide range of views, which included many negative and stereotypical **perceptions of people of Bangladeshi** origin as ignorant, fatalistic, and resistant to healthy lifestyle education.<br><br>Some wrongly believed that this population **view obesity as attractive** and a sign of wealth.<br><br>While healthcare professionals held many inaccurate perceptions about people of Bangladeshi origin, they also **willingly acknowledged their ignorance of Bangladeshi culture** and the Muslim religion, and called for **more training in cultural awareness**<br><br>**Healthcare professionals may have negative stereotypes of Bangladeshi people and consider them resistant to lifestyle advice:**<br><br>"I know that in Bangladesh the cultural norm is actually that to be of a heavier build is actually a sign of wealth so that's a cultural issue you have to keep in mind really because they might be quite resistant to taking on information about, you know, it's healthier to be thinner when they've got these kind of ingrained beliefs about weight and especially for men I think and boys because it's a sign of a wealthier family." [dietician, female]<br><br>**Religious leaders are widely respected and could play a key role in diabetes prevention education:**<br><br>"… because Ramadan is coming up again, and the Iman has such an influence over them. They have been saying to me the Iman says I can do this, I can do that, and if it's giving them the right message then you know, it's quite good, more than I would [be] telling them." [nurse, female]

S2: Extraction Tables

Study details	Population and setting	Methods & Study Quality	Findings
**Author**: Helmink<br><br>**Year:** 2012<br><br>**Setting / country**: The Netherlands<br><br>**Aim of study:** To examine factors explaining motivation among health care professionals to implement a multidisciplinary primary care-based lifestyle intervention<br><br>**Study design**: qualitative (in-depth interviews) and quantitative data (questionnaires)<br><br>Part of study called BeweegKuur, to support prevention and treatment of type 2 diabetes mellitus.<br><br>**Funding**: Dutch Ministry of Health, Welfare and Sports	**Number of participants**:<br><br>18 practices<br><br>**Pilot interviews:** General practitioners (n=2), practice nurses (n=3) and physiotherapists (n=4). All working in a primary care setting<br><br>**First questionnaire:** 59 health care providers (15 GPs, 25 physiotherapists and 16 practice nurses)<br><br>**Second questionnaire (Follow up from first):** 35 professionals completed (7 GPs, 16 physiotherapists and 12 practice nurses)<br><br>**Mean Age**: 42.2 years<br><br>**Gender:**<br>63% women (first questionnaire)<br>No data for second questionnaire<br><br>**Other**:<br><br>59% of the respondents were working in a primary health care centre, while 41% worked in a GP or physiotherapy practice.<br><br>**Intervention:** The BeweegKuur intervention is a 12-month GP practice intervention is to guide participants in achieving a sustained healthy lifestyle.<br><br>The intervention is intended for people with an impaired fasting glucose and people with type 2 diabetes. 22 patients participating per practice. Coaching and supervision are provided by a lifestyle advisor (practice nurse) based on principles of motivational interviewing. All medical specialists are offered training in motivational interviewing.<br><br>The lifestyle advisor designs an [illegible]	**Data collection methods**:<br><br>The qualitative data were gathered through in-depth interviews with health care professionals. Quantitative data collected by 2 questionnaires by mail.<br><br>Two measurements: baseline measurement at the start of intervention and second measurement 6 months later,<br><br>**Data Analysis**:<br><br>SPSS 15.0. Mean scale scores and standard deviations were calculated. Linear regressions were run using the backward deletion procedure ($P > 0.05$).<br><br>**Follow up**: response rate of 60% for both questionnaires<br><br>Dropout analyses showed that gender, age, working in a health care centre nor profession predicted response at the second measurement.<br><br>**Study Quality:**<br><br>Aims and objectives clearly stated and mixed methods<br><br>Chosen methods were suitable, but poor reporting of qualitative data<br><br>Flaws in study due to small sample size and lack of rigour in qualitative data.<br>No qualitative analysis/ results reported and this takes away from the overall message of the paper and does not correspond to study aims	**Main Themes relevant to research question**:<br><br>**Motivation for intervention**<br>Health care providers were motivated to implement the intervention (mean 7.84 on a scale ranged 1–10; SD 1.30). A total of 3.4% of the respondents indicated that their motivation was insufficient. At baseline, most respondents were also positive about their motivation to continue the implementation after the pilot period, with a mean motivation score of 7.79 (SD 1.29; range 1–10). The follow-up measurement also showed a generally positive motivation to continue the implementation (mean 7.84; SD 1.24; range 5–10).<br><br>**Characteristics of the socio-political context**<br>72.9% of the respondents felt supported by the colleagues within their practice. More than half (64.4%) felt supported by colleagues in their professional association and 66.2% by the institute that designed and implemented the intervention. More than a third of the health care professionals (39.0%) did not feel supported by their professional association.<br><br>**Characteristics of the innovation**<br>Overall, 79.6% of the health care provider's perceived relative advantages of the intervention for their patients and 62.7% perceived relative advantages for themselves. Some respondents (13.5%) thought that the intervention was complex, while others (27.2%) did not share this opinion and 59.3% adopted a neutral position in this respect. Half of the respondents thought that the intervention was compatible with their current work, while most of the other respondents (45.8%) gave a neutral answer.<br><br>**Characteristics of the user**<br>The majority (61.0%) of the respondents had a positive attitude towards the intervention, and 70.2% perceived a high subjective norm to implement it. High self-efficacy was reported by 35.6% of the health care professionals, while the others (64.5%) had a neutral opinion about their self-efficacy.

S2: Extraction Tables

Study details	Population and setting	Methods & Study Quality	Findings
**Author**: Jallinoja<br><br>**Year:** 2007<br><br>**Setting / country**: Finland<br><br>**Aim of study:** To explore physicians' and nurses' views on patient and professional roles in the management of lifestyle-related diseases and their risk factors<br><br>**Study design**: Quantitative questionnaire which was part of larger study and programmes:<br><br>Guidelines Implementation Programme (VALTIT) which aims to adapt and implement the Finnish Current Care guidelines on metabolic syndrome The programme is part of the GOAL project ((Good Ageing in Lahti Region) aiming to promote primary prevention<br><br>**Funding**: Academy of Finland	**Number of participants**:<br><br>Physicians (n=59) and nurses (n=161) working in primary healthcare (Total n=220)<br><br>**Mean Age**:<br><br>Physicians 46 years<br>Nurses 45 years<br><br>**Gender**:<br><br>Physicians 52% male<br>Nurses 99% female<br><br>**Other**:<br><br>66% (n35) of the physicians and 46% (n73) of the nurses had at least 16 years of work experience<br><br>Physicians: 54% (n32) had the basic education of licensed medical doctor, 37% (n22) were specialists in general practice, and 9% (n5) were specialists in some other field.<br><br>Nurses, 61% (n98) were public health nurses, 26% (n42) were registered nurses, and 13% (n21) had some other education	**Data collection methods**:<br><br>Questionnaire was piloted. Distribution of questionnaires was based on employee lists healthcare centres.<br><br>The total response rate was 59% (physicians 53%, n59; nurses 62%, n161) and the total number of respondents was 220<br><br>**Data Analysis**: The results were presented in cross-tables. Statistical testing of differences between the frequency distributions of physicians and nurses was carried out using a chi-squared test.<br><br>**Study Quality:**<br><br>Objectives of the study were clearly stated, however, a mixed method approach using qualitative methods would be more suited.<br><br>There was a modest response rate (69%).<br><br>Results are not explicitly discussed as they were mainly presented in tables- not fully discussed so this limited the believability of the study.	**Main Themes relevant to research question**:<br><br>A majority of physicians (88%) and nurses (95%) agreed that **patients themselves must accept the responsibility for lifestyle-related decisions**. A majority of physicians and nurses considered that patients' **unwillingness to change** is always or nearly always a key barrier to treatment.<br><br>Patients' **insufficient knowledge of the risk** of the condition was much more seldom regarded as a barrier, an opinion most pronounced in respect of adult obesity.<br><br>A majority of physicians and nurses were of the opinion that patients must be assigned **responsibility for the self-care** of dyslipidemia, high blood pressure, and type 2 diabetes. Dieters more often than quitters were seen as i**n need of professional support** in their effort to make a lifestyle change.<br><br>A clear majority of both physicians and nurses considered that information **provision, and motivating and supporting patients** in lifestyle change are part of their tasks. However, only slightly more than half of these professionals estimated that **they have enough skills** in lifestyle counselling, and two-thirds considered that **they have been able to help many patients** to change their lifestyles. Two-thirds of the physicians and one half of the nurses reported that their **schedule is too hectic** to allow them to go into patients' life situations. Nurses more often than physicians reported that they felt **uneasy intervening** in respect of patients' weight or smoking. Nurses with fewer years of professional experience were more likely to report **having enough skills for lifestyle counselling** (70% vs. 43%, p0.001).

S2: Extraction Tables

Study details	Population and setting	Methods & Study Quality	Findings
**Author**: Liddy<br><br>**Year**: 2013<br><br>**Setting / country**: Canada (Ottawa)<br><br>**Aim of study:** examined the feasibility of implementing a pre-diabetes program into a primary care clinic in Ottawa, Canada<br><br>**Study design**: Quantitative and Qualitative (questionnaire)<br><br>**Intervention:** "An Ounce of Prevention" Healthy Lifestyle and Diabetes Program was adapted from best evidence clinical trials and uses educational tools developed by the Diabetes Prevention Program for long-term behavior change, relies on principles of self-management, is group based and includes an integrated exercise component. We used a multi-method evaluation approach and examined feasibility and practical implementation aspects such as space, staffing, recruitment and retention issues.<br><br>Implemented a 4-week group program followed by an additional 12 weeks of contact either by phone or e-mail thus totalling 16 weeks of support however enabling overlap between the sessions.<br><br>**Funding**: program funding	**Number of participants**:<br><br>74 participants<br><br>73% response (n=47) rate on the patient surveys<br><br>**Mean Age**: 63 years<br><br>**Gender**:<br><br>**Other**:	**Data collection methods**:<br>multi-method evaluation approach and collected data through meeting minutes, patient questionnaires and patient records.<br><br>**Data Analysis**: Thematic analysis was used to analyses patient survey responses<br><br>**Study Quality:**<br><br>Objectives and methods were appropriate.<br><br>This paper lacked details on participants as there was only demographic data for participants was age. This made it difficult to understand how these findings may be applicable to other setting/populations<br><br>The sample was small and there was possible response bias.	**Main Themes relevant to research question**:<br><br>Overall patient **satisfaction** of the program was high with 83% (n=39) of ratings over 4/5.<br><br>Comments related to **general satisfaction**: "so far it's been excellent" [ID 801]; "excellent course" [ID 807]; "the whole program has been helpful and excellent" [ID 620]. Patients highlighted the benefits of the group setting "need a group to get me going" [ID 924].<br><br>The **informal, relaxed atmosphere** within the classes was also commonly raised as a positive feature: "friendly, relaxed atmosphere" [ID 915];<br><br>Positive feedback given about the overall content "very informative" [ID 941]; "Very conducive to learning and sharing of information" [ID 932].<br><br>Patients remarked on specific **informational needs** related to diet, exercise and blood work. Patients had low **awareness of the Canada Food Guide** and appreciated learning about it: "How interesting to find out about the food guides" [ID 820]; "I liked going over Canada's Food guide" [ID 815].<br><br>Many commented on the value of learning about **reading food labels**: I liked "reading labels" [ID 818];<br><br>The integration of **exercise** was appreciated: "Like having the exercise component" [ID 803];<br><br>Some requested having **more exercise** and/or modify when the exercise component was offered within the class: "I would include moving or exercise at the beginning of each class" [ID 735]<br><br>Several suggested having **on site lab facilities** for any required blood work and/or ensuring blood work is completed prior to the first class: "It would be nice if blood work could be done on site"

S2: Extraction Tables

Study details	Population and setting	Methods	Findings
**Author**: Lin<br><br>**Year:** 2012<br><br>**Setting / country**: USA<br><br>**Aim of study:** to describe the development and initial feasibility testing of the Avoiding Diabetes Thru Action Plan Targeting (ADAPT) program to enhance counselling about behaviour change for patients with pre-diabetes.<br><br>**Study design**: Qualitative (interviews)<br><br>**Funding**: National Institute of Diabetes and Digestive and Kidney Diseases	**Number of participants**:<br><br>**Phase 1:** development of ADAPT intervention includes involvement of a multi-disciplinary panel and interviews with primary care providers (PCPs) (n=12) and patients (n=8)<br><br>**Phase 2:** Feasibility testing of intervention included a convenience sample of two PCPs and four of their randomly selected pre-diabetic patients<br><br>**Age**: range from 38-58 (phase 2 only. Phase 1 not reported)<br><br>**Gender:** 100% female patients and PCPs (phase 2 only. Phase 1 not reported)<br><br>**Other**: Providers had been in practice for 5–10 years.<br><br>**Intervention**: The ADAPT program combines a streamlined shared goal-setting tool embedded in the electronic health records (EHR) with elements derived from cognitive and non-cognitive behavior change theories to help PCPs more effectively counsel patients with pre-diabetes to improve lifestyle behaviors.<br><br>An interdisciplinary team of 6 members with relevant expertise developed the intervention. Next, in-depth interviews were conducted with PCPs and patients to evaluate their attitudes toward pre-diabetes and barriers to lifestyle changes. These data were used to guide the	**Data collection methods**: Primary care providers and patients were interviewed about their perspectives on lifestyle changes to prevent diabetes. Semi-structured interviews lasting 30 – 60 minutes<br><br>**Data Analysis**: Audio-recorded and transcribed for thematic elements. The initial coding was done by one investigator and a second investigator reviewed codes for thematic congruence.<br><br>**Study Quality:**<br><br>Objectives and methods were appropriate.<br><br>First phase did not report enough demographic data which made it difficult to apply these findings to other settings/populations.<br><br>Qualitative analysis was appropriate and findings were adequately described.	**Main Themes relevant to research question**:<br><br>**Phase I: development of the ADAPT program**<br><br>**Provider interviews**<br>Providers felt that diabetes was a significant problem in their patient population and estimated the prevalence of diabetes in their patient population at 20 – 40% (with another 30 – 50% at risk for developing diabetes). However, providers felt their patients who were at risk for diabetes may not understand their level of risk.<br>"I don't think [it] really impacts them…they don't really see it as an active issue" [female provider in practice for 7 years]).<br><br>Barriers to counselling included: lack of time, workflow disruption ("other factors, the focus of the patient…[other] distractions" prevent discussion [female provider in practice for 14 years]), low self-efficacy ("I have very little confidence in my ability to quantify somebody's risk" [male provider in practice for 6 years]) and low response-efficacy ("a lot of people just think 'well, how's that going to make such a big difference"' [female provider in practice for 14 years]).<br><br>Some techniques that providers felt were useful for counselling were the use concrete examples or goals ("I usually focus on what's the tangible, active issues so they can understand" [female provider in practice for 7 years] and "what has worked for me is small steps that I can discuss with the patient and I am going to re-visit on the next visit" [male provider in practice for 20 years]) and motivating patients by tapping into patients' concerns ("You just try to hook onto something that is important to them" [female provider in practice for 4 years]).<br><br>**Patient interviews**<br>Interviews with patients showed that most were aware of their high-risk status. Many had family members or knew friends with diabetes so knew about the types of foods to eat and diabetic complications.<br><br>Patients generally believed they could prevent diabetes by making lifestyle changes ("stop eating all that sweet, that sodas, that juices" [female, 23, African–American] or "work on my weight, exercise" [female, 62, white]).<br><br>They listed lack of time ("by the time you come home, you just want to lay down" [female, 44, Hispanic]), motivation ("I need that push" [female, 54, Hispanic]) and preference of unhealthy habits ("the changing completely of cooking and eating…eating what you're supposed to, not what you really want" [female, 62, Hispanic] and "you have to change everything, your whole lifestyle" [female, 38,

Study details	Population and setting	Methods	Findings
	development and refinement of a prototype of the intervention. The prototype was tested through usability studies with PCPs and further refined. Lastly, the ADAPT program underwent a feasibility study to further improve the program.		African-American]) as common barriers for behavior change.<br><br>Most providers liked that the electronic health record (EHR)-embedded goal-setting tool was simple and straightforward and thought they would use it if it did not disrupt their workflow.<br><br>The primary barrier was unfamiliarity with the tool. Furthermore, some screens were considered too text heavy and at times confusing. The alerting mechanism was a concern for its possible disruption of providers' usual workflow.<br><br>**Phase II: feasibility testing of ADAPT program**<br>The providers reported that the goal-setting tool helped focus their pre-diabetes counselling and led to shorter but more effective counselling. Providers reported that using the EHR-embedded goal-setting tool took less than 5 min and that counselling took 5–10 min total.<br><br>Providers noted positive reactions from their enrolled patients who appreciated the "behaviour change samples" and website reinforcement.<br><br>At baseline, the average A1C was 5.6 and at 3 months remained at 5.6. Patients' exercise goal to increase number of steps per day ranged from 500 to 2000 extra steps. Two patients chose the diet goal to "use a 9-in. plate" for meals, one chose to reduce number of sweetened beverages and one chose to eat out less often each week. Average baseline steps per day measured by the pedometer were 4284 and increased to 5250 at 3 months. Some participants who responded to the tailored reminders wrote comments about how they were doing including "I have lost five pounds" and "Sorry I didn't really carry the meter this week. Had a tough week. I will be more focused this week."

S2: Extraction Tables

Study details	Population and setting	Methods & Study Quality	Findings
**Author**: Lu<br><br>**Year:** 2013<br><br>**Setting / country**: Australia (New South Wales)<br><br>**Aim of study:** to explore patients' views on risk, assessment and their general practitioner's role, and how these factors may impact their uptake of preventive care.<br><br>**Study design:** Qualitative (semi-structured telephone interviews)<br><br>Sub-study of a randomised controlled trial of preventive care in general practice, the Preventive Evidence into Practice (PEP) study.<br><br>**Funding**: National Health and Medical Research; Council, The Royal Australian College of General Practitioners, the National Heart Foundation of Australia and the BUPA Foundation.	**Number of participants**: 18 patients from three general practices<br><br>**Age**: Age range from 40-69 years<br><br>**Gender**: 11/18 female patients	**Data collection methods**:<br><br>Semi-structured telephone interviews<br><br>The study population was drawn from three New South Wales general practices involved in the intervention arm of the trial.<br><br>Interviews lasted for an average of 18 minutes (range 8–33 minutes).<br><br>**Data Analysis**: Qualitative thematic analysis from transcribed interview scripts.<br><br>**Study Quality:**<br><br>Objectives and method were appropriate, but reporting brief in parts resulting in an inadequate exploration of the data.<br><br>Authors stated saturation reached (18 patients) which helps to combat biases in reporting.	**Main Themes relevant to research question**:<br><br>**Patient views on risk**<br>Patients perceived lifestyle habits (diet and exercise), weight, family history, blood tests, or physical examination results as the main factors influencing their own risk of heart disease and T2DM. They appraised these individual risk factors differently according to whether overall they felt at a higher or lower risk. Patients who felt at risk of either or both diseases commonly cited their family history for the disease as the main reason.<br><br>'Maybe heart disease, not diabetes … I feel the family history probably qualifies me for some problem later on.' [P 01]<br><br>Patients who felt they were not at risk of the diseases interpreted their risk profile as a combination of lifestyle, absence of family history and other factors.<br><br>'I pretty well look after my health and I exercise regularly and I eat the correct food most of the time. I go to the doctor probably once every 12 months for a thorough check-up … I can't see anything else. It doesn't run in my family.' [P 07]<br><br>Some patients expressed uncertainty about their risk profile because of a disjunction between individual risk factors, some of which they felt put them at higher risk and others that reflected a lower risk.<br><br>'Maybe not. Because I've always had a lot of sugar and chocolates ... but I've been fine every time, yeah I've been tested. So I'm not sure whether it makes a lot of difference or not.' [P 15]<br><br>**Patient's views on risk assessment**<br>Patients' views on risk assessment were influenced by what they experienced in general practice. All patients reported having blood tests performed by their GP to assess their blood sugar and cholesterol levels. Some also described undergoing physical examination such as weight measurements and blood pressure checks. No patients interviewed recalled having had multifactorial risk assessments for cardiovascular disease or diabetes.<br><br>In most cases, the blood tests were conducted as part of routine check-ups done regularly, at least once in the previous 2 years. A few patients reported having blood tests for sugar or cholesterol levels after presenting with risk factors (eg. high blood pressure) or symptoms. The majority of patients had a positive attitude to taking blood tests, preferring to know their risk.

Study details	Population and setting	Methods & Study Quality	Findings
			'I just think it's important because it's something they say can be a silent killer if you don't know that you have high cholesterol.' [P 15]<br><br>One patient had a negative attitude to blood tests, believing these were only warranted if/when she became symptomatic. As the carer for her husband who has T2DM and heart disease, she was confident of being able to recognise symptoms.<br><br>**Patients views on the role of their GP in preventive care**<br>Patients described three distinct roles for GPs in helping them stay 'healthy' and 'disease free':1. providing check-ups and blood tests 2.monitoring their lifestyle 3. giving advice, both addressing present concerns and preventive.<br><br>The former two roles reflect the patients' views of the GP as a source of monitoring and support to their own efforts to lower risk. Those patients who emphasised 'role 3', tended to view prevention as a personal responsibility, independent of GPs.<br><br>Patients' actions were linked to the way they used their GP. Patients who regularly attended their general practice for check-ups were more likely to discuss general health and lifestyle and see the provision of preventive advice as part of their GP's role.<br><br>'I think it's important that he keeps up-to-date with the latest preventive things that are available, or information or provide me with guidance.' [P 07]<br><br>Patients who only consulted their GP for specific problems were more likely to receive reactive care focused on that issue alone. Some of these patients preferred this approach. Others nonetheless wanted their GP to initiate check-ups and broader discussions on health.<br><br>'I think they have an enormous responsibility to actually bear on their patients to [start] living in a healthy manner.' [P 02]<br><br>**Patient action**<br>The preventive action taken by patients was predominantly lifestyle related, reflecting advice given to them and their perception of its importance. All patients described taking physical activity or dietary measures (commonly both) to lower their risk for diseases and some aimed to achieve weight loss through such efforts. Their perception of 'risk' was inherently linked to their motivation to take up preventive actions. When it came to following their GP's advice, patients were motivated by their blood test results. The response of patients with 'bad' blood test results ranged from being 'not concerned' to 'shock', 'concern' and

Study details	Population and setting	Methods & Study Quality	Findings
			disappointment'.<br><br>The main difficulty with maintaining or making positive lifestyle changes was time constraints due to work or family commitments. Many patients would have liked to increase their physical activity if they had the time.<br><br>'It leaves me very little time after work … to indulge in any physical activity on a regular basis.' [P 02]<br><br>Cost was a related barrier for some patients to continue to participate in lifestyle programs.<br><br>'Cost probably was 'cause I used to go to a gym and I was working and it was getting expensive as well and I wasn't getting there very often, so yeah I stopped going.' [P 15]<br><br>Patients also cited stress and difficulty breaking old habits as barriers to maintaining positive lifestyle changes. Stress caused some patients to revert to unhealthy eating patterns. Older patients found it difficult to maintain their lifestyle changes because their 'bad' habits were so ingrained.<br><br>'You have to change the pattern, you have to change the lifestyle … It might be difficult. I think when you grow old, you are so set in your ways and you don't like change.' [P 13]<br><br>**The patient-doctor relationship**<br>Central to the interaction was the relationship patients have with their GP. Trust was bolstered by GPs' professionalism, knowledge, patience, honesty and willingness to address patient concerns. Patients preferred it when their GP gave clear explanations and presented a range of choices so they could make informed decisions on their own health. Patients recognised that GPs gave 'sensible' advice 'for good reason'.<br><br>'But I tend to follow, the advice of the doctor is for a good reason and it's, and I would be stupid if I didn't take the advice of the doctor.' [P 06]

S2: Extraction Tables

Study details	Population and setting	Methods & Study Quality	Findings
**Author**: Schütze<br><br>**Year:** 2011<br><br>**Setting / country**: Australia (New South Wales)<br><br>**Aim of study:** to determine the feasibility of lifestyle modification programs for disease prevention in general practice<br><br>**Study design**: Qualitative (interviews)<br><br>Part of a cluster randomised controlled trial, called HIPS, assessing prevention of vascular disease in patients<br><br>**Funding**: National Health and Medical Research Council<br><br>**Key:** lifestyle modification programs (LMP) practice nurses (PNs) and allied health (AH)	**Setting**:<br>13 general practices<br><br>**Number of participants:**<br>11 general practitioners<br>4 practice nurses<br>5 allied health providers<br><br>**Age Range**:<br><br>GPs: 35-34 years<br>Nurses: 25-64<br>Allied professionals: 25-44 years<br><br>**Gender**:<br><br>GPs: 5/10 females<br>Nurses: 3/5 females<br>Allied professionals: 3/5 females<br><br>**Other**:<br><br>**Years of experience (range):**<br><br>GPs: 15-39 yrs.<br>Nurses: 2.5-20 yrs.<br>Allied professionals: 3-10 yrs.<br><br>**Intervention:**<br><br>The HIPS intervention aimed to assist high risk patients make positive lifestyle changes by providing a brief intervention tailored to patients' stage of change.<br><br>This was done as part of a health check in general practice, with referral of high risk patients to a LMP coordinated by trained facilitators.	**Data collection methods**:<br>Interviews with general practices in the intervention arm of this trial examined their views on implementing the lifestyle modification program in general practice settings.<br><br>**Data Analysis**: thematic analysis. Codes developed and verified by research theme. Use of Nvivo. The coding frame was developed based on a mixed deductive and inductive approach.<br><br>**Study Quality:**<br><br>This study was the qualitative component of a RCT.<br><br>Objectives, methods, and data collection and analysis were appropriate.<br><br>This study utilised volunteers so subject to selection bias, but this is not uncommon for this type of study.	**Main Themes relevant to research question**:<br><br>**<u>Fit with general practice routines:</u>**<br>GPs reported that offering brief advice regarding lifestyle modifications were quite **easy to incorporate as part of a health check consultation**. Many GPs reported that this was already part of their normal practice and some felt that they were already proactive with preventative lifestyle measures for their at-risk patients. They reported previously offering advice, providing printed material, and arranging referral for at least some of their patients.<br><br>The **training and practice support** provided to GPs and AH providers as part of the project allowed a more systematic and planned approach to preventive care.<br><br>"We already were doing quite a lot of ... those things in a lot of patients. . . So we were oriented towards that already anyway". (GP #2)<br><br>One GP suggested that it would have been better to have **more materials on the computer** to print out and an electronic form to aid in the assessment of patients. Some suggested the use of a **standardised form would aid monitoring and follow up**.<br><br>"It'd be easier on review to have a standardised form, the same as in the initial assessment ... to be able to repeat that process ... at the follow-up visit would actually have been really useful and they could have got the original one out, compared it ... with the next one and made direct comparisons with the patient: 'Look you know you were drinking 20 drinks a day and now you're down to four.' (GP #1)<br><br>Some respondents appreciated the recall of patients to the practice for a **specific health check visit,** especially because this encouraged the more motivated patients to present. Others felt health checks should be performed on all their patients to allow earlier detection and prevention of chronic disease.<br><br>"It links in very well with the fact that we should be doing our health checks on everybody that walks in here. . . So that you can pick up things before they get to a stage. . . "(PN #1)<br><br>**<u>Communication with the AH providers and group program:</u>**<br>Most GPs found referral to be easy; however, this was dependent on the quality of

Study details	Population and setting	Methods & Study Quality	Findings
	The LMP comprised two AH visits (at the AH provider's practice) and four group sessions of 1.5 h each over a three month period with follow-up review sessions at six and nine months. Each group session included an educational component as well as a physical activity component consisting of 20–30 min of walking or resistance exercise.		organisation provided by individual Division coordinators. Several respondents felt that the relationship of the practice with the Division coordinator was critical to the success of the program.<br><br>The **major criticism was the lack of feedback** from the AH provider to the GP. This disappointed most GPs as they wanted to know whether the patient had been to see the AH providers and what goals had been set so that they could provide on-going encouragement.<br><br>**<u>Perceptions of their patients' experience:</u>**<br>Respondents reported that their patients had relayed very **positive experiences** of the LMP, and GPs perception was that their patients had gained health literacy and awareness.<br><br>GPs felt that this was more **structured and detailed** than they were able to provide in brief consultations and also reinforced the health messages that they gave in the consultation.<br><br>"You know usually when I see the patient, I, I tell them what to do. But I don't, I don't cook for them. I don't order meal for them ... if I refer patient to a dietician or exercise physiologist or something like that ... they don't give the patient the practice ... I advise the patient you, you, you give the practice, the practical part of what I'm telling patients ... make my advice become real". (GP #8)<br><br>Respondents reported that they felt that the **realistic goal setting** that was encouraged in the LMP was important to helping patients make lifestyle changes as was the group support patients received from peers in the LMP.<br><br>Respondents reported that their patients **engaged with the advice given** and this helped to motivate and support them to make lifestyle changes. The LMP provided encouragement for patients to take a more active role in their own health care.<br><br>"I found that people who are ready to change took the info and did the interventions [and] were motivated. Others did not, but most made some small change, which is positive, anything is better than nothing. Some have readily taken exercise on board." (PN #2)

S2: Extraction Tables

Study details	Population and setting	Methods & Study Quality	Findings
			The **biggest barrier to accessing the program** both in the urban and rural areas was transport. For patients who worked or had family commitments, the availability of the program out-of-hours was also an issue.<br><br>"... if these people are working they can't unless it's delivered in the hours that are suitable ... you're talking about you know targeting people in their 30s, 40s, 50s and they're still working." (GP #7)<br><br>Several respondents reported that **cost would also have been a barrier** had the group program and AH visits not been provided free of charge to the patient.<br><br>**<u>Sustainability of implementing LMPs in primary health care:</u>**<br>The major **barriers reported to greater implementation** in general practice were **time and competing priorities** with other health issues, especially in older patients with existing chronic illness.<br><br>Providers reported that they were able to **incorporate risk factor assessment** more routinely and comprehensively than previously because the focus of the consultation was the health check and therefore they had more time for it.<br><br>"... this was, you know, a deliberate one, a consultation, which was specifically catered to their, umm, problems from that end." (GP #6)<br><br>**Practice nurse involvement** was seen as an important factor in long-term sustainability and effectiveness of the LMP as they were able to carry out many of the initial and follow-up assessment measurements and offer brief advice tailored to the patient's readiness for change.<br><br>"... a lot of patients, before they see the doctor they see the nurse first who'll take their smoking history, their alcohol history, measure their weight, will offer brief intervention."(GP #1)<br><br>Respondents felt that there was a need for preventive services for people at risk of developing chronic disease and these were **currently lacking in the health system.**<br><br>"And there are a lot of things available for people with conditions... Not so much for ones who are trying to be proactive." (GP #7)

S2: Extraction Tables

Study details	Population and setting	Methods & Study Quality	Findings
			<u>**Sustaining lifestyle changes**</u><br>The challenge for patients in **maintaining their lifestyle changes** was raised by many respondents. **Continuity of care** was a major factor influencing how successfully the LMP could be sustained and how well patients maintained their lifestyle changes.<br><br>Few respondents felt that their patients would maintain their lifestyle changes **without long-term follow up** and that services were required to support this.

S2: Extraction Tables

Study details	Population and setting	Methods & Study Quality	Findings
**Author**: Troughton<br><br>**Year**: 2008<br><br>**Setting / country**: UK (England, Midlands)<br><br>**Aim of study:** to inform the development of an educational intervention for people with pre-diabetes in the UK<br><br>**Study design**: Qualitative (interviews)<br><br>**Funding**: University Hospitals of Leicester NHS Trust Research Fellowship award	**Number of participants**:<br><br>15 diagnosed with pre-diabetes from the community as part of a screening programme.<br><br>**Mean Age**: 57 years<br><br>**Gender:** 47% were female<br><br>**Ethnicity:** 60% were White European and 40% were South Asian	**Data collection methods**:<br>The development of a topic guide and two pilot interviews. One-to-one, semi-structured interviews. Interviews lasted between 30 and 45 min<br><br>**Data Analysis:** framework methodology. interviews were coded using the QSR n6 (NUD × IST) qualitative data-indexing package<br><br>**Study Quality:**<br><br>Objectives, methods, and data collection and analysis were appropriate.<br><br>This study provided a rich account of participant experiences. Authors claimed saturation was reached (n=15)	**Main Themes relevant to research question**:<br><br>**<u>3.1. Seriousness of pre-diabetes</u>**<br>Uncertainty about the seriousness of pre-diabetes was a strong theme throughout all of the interviews. These views were influenced by a variety of factors; prior experience of diabetes and pre-diabetes, knowledge of pre-diabetes, receiving the results, perceptions of responsibilities, and lack of planned follow up and healthcare professional beliefs.<br><br>**<u>Prior experience of diabetes and pre-diabetes</u>**<br>No participant had any experience of pre-diabetes prior to his or her own diagnosis; "*I've never heard of this Pre Diabetes stuff*".<br><br>However all (*n* = 15) participants had experience of friends or family members with diabetes. Diabetes was considered to be **serious with issues** such as injections, hypoglycaemia and a loss of independence as commonly cited fears. These concerns were often explicitly related to the participant's perception that those close to them had experienced adverse consequences from diabetes.<br>"*You can go into comas if your blood goes down…*"<br><br>**<u>Knowledge vacuum</u>**<br>For all participants, lack of knowledge about pre-diabetes created a vacuum and made it difficult for them to evaluate the meaning and seriousness of their diagnosis. *"the letter had the tests they did, with the results… not that I really understood them"*.<br><br>**<u>Identity</u>**<br>For most (n = 10), pre-diabetes was seen as a grey area, which led to uncertainty about the meaning of pre-diabetes, and the seriousness of the condition.<br>"I am borderline diabetic, I'm in that grey area, not quite diabetic but I could be".<br><br>For many (n = 8) identifying that they had a diagnosis of pre-diabetes was made more difficult and confusing by being free of symptoms.<br>"You're not suffering any physical symptoms that you would say I really need to see the doctor right now".<br>3.1.2.2. Causes<br>Although most (*n* = 13) participants could give reasons why a person might develop pre-diabetes, many (*n* = 8) participants struggled to see why it had affected them.<br><br>**<u>Consequences</u>**<br>Most (n = 10) respondents were unclear how risk applied to them and how such a diagnosis had been made.

S2: Extraction Tables

Study details	Population and setting	Methods & Study Quality	Findings
			"I don't know what they found to make them think I am at risk in the future… what would make them believe that I will develop diabetes. I don't know why?"<br><br>For many (n = 8) it was important to be able to understand how the risk had been determined.<br>"everyone is at risk, knowing why you are at risk is really important".<br><br>The uncertainty of the future was a concern for many (n = 11) but was talked about more often by all those of South Asian origin (n = 4<br><br>There was a wide range of opinion if and when diabetes might develop,<br>"you might get diabetes but then again you might not"<br>"all of a sudden you could be dead"<br><br>**<u>Receiving the results</u>**<br>The longer the results letter took to come; the diagnosis was viewed as less serious.<br>"*When you don't get that call in a day or two, you think, well it can't be all that bad*".<br><br>**<u>Whose responsibility?</u>**<br>Once the results letters had been received some (*n* = 5) participants felt that it was the GPs responsibility to contact them and when they were not contacted, this enhanced the view that pre-diabetes was not serious.<br>"*If I was really at risk they would have called me*" and N13 "*I just assumed everything was fine and that is why they didn't send for me*".<br><br>**<u>Health care professional beliefs</u>**<br>For those that did contact a professional (*n* = 4), the perceived professional's behaviour and comments clearly influenced how the respondent viewed the gravity of their condition.<br>"*when the GP said this was serious, then I knew it's not funny or to treat lightly*<br>"*I mentioned it to my doctor and he said 'there's really nothing to worry about*".<br><br>**<u>Taking action</u>**<br>During the interviews all participants (*n* = 15) reflected on their diagnosis and how they dealt with it. Whether to act on the diagnosis of pre-diabetes or not was influenced by many factors; uncertainty about preventative action, lack of planned follow up, written information and support.<br><br>**<u>Uncertainty about preventative action</u>**<br>Most respondents (*n* = 10) expressed concerns about how to take preventative action and were uncertain what to do.

S2: Extraction Tables

Study details	Population and setting	Methods & Study Quality	Findings
			"*I want to prevent it if I can, and I don't know how. I am up in the air and hoping*".<br><br>This lack of knowledge was, for some, accompanied by explicit cost–benefit analyses and a concern around legitimacy of lifestyle change, its magnitude and efficacy.<br><br>**<u>Lack of planned follow up</u>**<br>Most (*n* = 12) were disappointed by the lack of planned follow up, "*I feel left in mid-air to fend for myself*".<br><br>A common consequence of lack of knowledge and uncertainly about the seriousness of pre-diabetes, was overt distress, which appeared more pronounced if a plan for subsequent re-screening and follow up was unclear.<br>"*You've got me so far down the path and left me, and I have a number of paths to choose now. I don't know which one to choose and I might just walk back. It is not satisfactory to screen people and not follow them up properly*".<br><br>**<u>What more can I do?</u>**<br>A few (*n* = 2) respondents (both Asian) passively sought solution in medication, N5 requesting "*tablets available for pre-diabetes, to prolong it*".<br><br>Some (*n* = 5) respondents believed that they were already judicious in their self-care that they already had made reasonable lifestyle changes and did not understand what further changes they could make.<br>"*I know all these things and I've been doing these things… so what else do you want me to do*".<br><br>**<u>Written information</u>**<br>Although the respondents had all been issued with an information booklet about pre-diabetes upon diagnosis it was felt by most (*n* = 12) not to be useful,<br>"*I just had a basic booklet… it didn't help to be honest*", N2 "*the information was too vague*", and N5 "*it was just the same for everybody*".<br><br>**<u>Support</u>**<br>All (*n* = 15) participants would have liked to have been seen a healthcare professional shortly after diagnosis,<br>"*the letter did explain it, but I think if somebody actually called you in and said we've just had these results, and this is what you need to do, and why you need to do it; it would have helped*".<br><br>Throughout interviews were pleas for support to deal with the diagnosis, and more particularly the consequences of anxieties and behaviour change. Most participants

S2: Extraction Tables

Study details	Population and setting	Methods & Study Quality	Findings
			(*n* = 12) reported discussing their diagnosis with friends and family who themselves had diabetes, perceiving this to be a valuable source of factual material.<br><br>Respondents also noted the need for consistent information and coherent support from their health providers<br>"*the GP said sort of stick to unrefined carbohydrates and I said 'what are unrefined carbohydrates' and he said go and see the practice nurse*'. *What got to me was that she said absolutely no carbohydrate*".<br><br>Most (*n* = 13) participants expressed a wish for structured information and regular, responsive contact with a healthcare professional to help make lifestyle changes.<br>"*I want to be told by someone who knows*"<br>"*I want more contact with you to keep a check on me*. *If you know you have a check you tend to stick to a diet better. I find dieting really hard*," and N3 "*If they consider I am high risk through family then it gives you confidence to think they are keeping an eye on you*".<br><br>A few (*n* = 3) participants, particularly those who actively sought information on pre-diabetes, were convinced that there should be a specialist support service available for people with pre-diabetes comparable to other areas of care,<br>"*The only preventative medicine that we do in this country seems to be family planning*. *I think if there was a diabetic equivalent of the family planning service that would be good*".<br><br>Many participants (*n* = 7) could see the benefits of attending group education,<br>"*groups can support you more… having people with you that are the same way*, *you find you're not on your own fighting against things*".<br><br>All participants placed high value on a repeat test for pre-diabetes which would reassure them they were on the right tracks regarding the lifestyle changes made.<br>"*It lets me know if I am doing right or am I doing wrong*"<br>"*If it's creeping up, I know I have to do something more*".

S2: Extraction Tables

Study details	Population and setting	Methods & Study Quality	Findings
**Author**: van Esch<br><br>**Year**: 2013<br><br>**Setting / country**: The Netherlands<br><br>**Aim of study:** to explore Dutch health care professionals' attitudes regarding current or future uptake of a more extensive use of FH information and the family system in diabetes prevention.<br><br>**Study design**: Qualitative (interviews)<br><br>**Funding**: Dutch Diabetes Research Foundation and University Medical Center	**Number of participants**:<br><br>19 health professionals (from 6 general practices, and 4 outpatient diabetes clinics<br><br>**Mean Age**: not reported<br><br>**Gender**: 58% female<br><br>**Ethnicity**: All interviewees were of Dutch origin<br><br>**Experience in practice (n):**<br>0-10 years (10)<br>11-20 years (4)<br>21-30 years (3)<br>> 31 years (2)	**Data collection methods**:<br>Purposive sampling strategy. General practices were recruited via the regional network. Semi-structured interviews were conducted with nineteen health care professionals.<br><br>**Data Analysis**: All interviews were digitally recorded, transcribed. Qualitative data indexing software (ATLAS.ti 5.2) was used for data coding and retrieval. Analysed using thematic content-analytical techniques.<br><br>**Study Quality:**<br><br>Objectives and methods were appropriate.<br><br>Poor reporting of demographic data as mean age of participants was missing.<br><br>Data was based on a small sample of 15 with no indication of saturation so it was difficult to judge if the sample size was adequate.	**Main Themes relevant to research question**:<br><br>**<u>Mapping current practice</u>**<br>All included GPs work in accordance with the Dutch guideline for diabetes treatment, which include opportunistic screening for T2D.<br><br>All interviewees perceive FH (Family History) (specifically in first-degree relatives) as important factor in the risk stratification procedure. However, according to the interviewees in primary care, the assessment of FH information is not standardized; a person's FH of diabetes is inquired the moment it is thought to be of relevance.<br><br>Professionals vary in asking about second-degree relatives with T2D. Both in primary and secondary care, FH information is registered in electronic medical records (EMRs), but not with a retrievable code.<br><br>When patients are diagnosed with (pre)diabetes, they are regularly monitored and receive education about T2D risk factors and lifestyle modifications to prevent diabetes complications.<br><br>**<u>Using family history information in preventive actions</u>**<br>Data revealed that the extent to which the multifactorial aetiology of T2D is explained varied between professionals: *'It depends on the patient, whether (s)he is interested. But I try to explain that some people are more at risk than others.'* [GP4].<br><br>Some GPs and PNs do not emphasize the role of FH as a risk factor, as it is not a factor that can be changed: *'We think monitoring weight and blood glucose levels in this population is most effective. We don't emphasize family history.'*[GP3] *'Understanding the balance between food consumption and energy expenditure, that's what counts.'* [GP5].<br><br>None of the interviewees used FH information to promote health-protective behaviour. The clarification of what FH could mean to a person by professionals seems to be minimal. Professionals could not bring up absolute or relative risk estimates of developing T2D in persons with a FH. Nevertheless, they agreed that personal perceptions about diabetes running in the family could be discussed more thoroughly and knowledge about familial susceptibility to diabetes could be improved: '<br><br>The idea of using FH information to motivate risk-reducing behaviour was new to all interviewees, but it was acknowledged that for some relatives, personalized risk messages could be a cue to action: *'I think, targeting family members could be*

S2: Extraction Tables

Study details	Population and setting	Methods & Study Quality	Findings
			effective. However, I think you should reach them in a neutral and thoughtful manner. People don't want you to interfere with their personal life too much.' [GP2].<br><br>All primary care professionals reported to be interested in new strategies and tools to inform people about the importance of a healthy lifestyle. None of the interviewees had paid attention to or used the information provided by renowned Dutch health organizations.<br><br>**<u>Directly targeting patients at familial risk developing type 2 diabetes</u>**<br>Most GPs and PNs indicated that directly targeting and educating populations at risk, including persons with a FH, would be desirable and worthwhile:<br>*'We plan to set up more preventive activities targeting patients with an extensive family history of cardiovascular disease and type 2 diabetes.'* [GP4].<br><br>However, they foresee practical problems; lack of time, finance and organizational barriers were reported: '*What we need is a continuing approach. Our PNs are trained to provide patient education and motivate patients in the process of behaviour change. […] We could organize and facilitate a structured programme, on condition that financial resources are available.'* [GP2].<br><br>Most importantly, however, directly targeting patients with a FH is not possible because electronic medical records are not equipped to retrieve persons with a FH.<br><br>**<u>Asking patients to pass on risk and preventive messages in their family</u>**<br>The idea of asking patients to inform relatives about familial susceptibility to T2D appeared to be new to all interviewees.<br><br>During the interviews, the professionals became more and more interested in this potential approach to reach relatives at risk: *'When you think about prevention, you have to reach as much people as possible. I do not disapprove this kind of targeting approach.'* [PN4&5].<br><br>Interviewees referred to patients who bring up inheritance and concern about the future health of their relatives themselves. They commended on the possibility of contacting otherwise unreachable healthy individuals and thought that a specific group of patients seems willing to disseminate information in their family: *'Patients who adequately handle their disease will be motivated to participate. Other patients are into denial and/or struggling with their disease. You can't ask these patients to deliver diabetes risk messages in their family.'* [PN6a].<br><br>However, for some GPs it was difficult to think about targeting a population that

S2: Extraction Tables

Study details	Population and setting	Methods & Study Quality	Findings
			does not necessarily include their own patients: *'I think it is difficult to manage, sometimes I ask about relatives, but most relatives are not registered as a patient in our practice.'* [GP1]. Moreover, besides a lack of time during their consultations, they indicated that they would need expertise and skills to guide and educate patients who are willing to serve as a messenger in their family.<br><br><br>**<u>Perceived barriers regarding a patient-mediated targeting approach</u>**<br>Some questioned the feasibility and benefits of a patient-mediated approach in diabetes prevention: 'Patients don't want to deliver bad news and relatives don't want to receive such messages.' [DS3a].<br><br>They doubted whether patients would be able to pass on accurate messages and whether relatives will be alarmed:<br><br>Different professionals mentioned strong family bonding in ethnic minority families as a potential advantageous factor: 'Family ties seem to be stronger in immigrant families.' [DS3].<br><br>Conversely, other professionals emphasize cultural and linguistic barriers. Some GPs and PNs do not expect benefits from illuminating the familial character of T2D in ethnic minority groups because of differences in perceived controllability with regard to health and illness, causal attributions, generational conflicts and low literacy: 'The illness burden of first generation migrants might not impress the younger generations. These youngsters do not identify with their parents as far as health-related issues are concerned.' [GP5].<br><br>Asking non-Dutch patients to pass on information seems not feasible according to these professionals: 'It's the other way around. Those children are used to translate during consultations and provide their parents with health information. They won't listen to their parents and will search for information themselves when they need it.' [GP4].<br><br>Moreover, the younger generation in general would not be admissible to risk messages via the family system: 'I question whether it's effective. Younger offspring is not concerned with future health risks.' [GP1] 'Do children listen to their parents? […] I think a person will be interested the moment (s)he is confronted with the problem.' [DS3a].

S2: Extraction Tables

Study details	Population and setting	Methods & Study Quality	Findings
**Author**: Vermunt<br><br>**Year:** 2012<br><br>**Setting / country**: Netherlands<br><br>**Aim of study:** to explore opportunities for refining intervention delivery.<br><br>**Study design**: Quantitative (questionnaire)<br><br>Part of trial: APHRODITE lifestyle intervention for the prevention of type 2 diabetes in Dutch primary care.<br><br>**Intervention:**<br>A 2.5-year intervention was performed in 14 general practices in the Netherlands among individuals at high risk for type 2 diabetes (n = 479) and was compared to usual care (n = 446).<br><br>Intervention consisted of individual lifestyle counselling by nurse practitioners (n = 24) and GPs (n = 48) and group-consultations.<br><br>**Funding**: The Netherlands Organization for Health Research and Development	**Not clearly reported in article. Estimates derived from piecing information together from trial data.**<br><br>**Number of participants**:<br><br>GPs: n=48<br>Nurses: n= 24<br>Patients from intervention arm: 479<br><br>(numbers estimated given dropout rates because information was not provided in article)<br><br>**Mean Age:** 58 year (patients)<br>Health practitioners not reported<br><br>**Gender**: 61% female (patients) (baseline data). Health practitioners not reported<br><br>**Other**:	**Data collection methods**:<br><br>Questionnaires were developed and reviewed by an expert panel of epidemiologists, GPs and nurse practitioners.<br><br>**Provider questionnaires** were filled out within one month after finishing the project.<br><br>Response to the questionnaires was 80 % within GPs and 100 % within nurse practitioners.<br><br>**Participant questionnaires** (intervention group) were filled out during the 30-month data collection.<br><br>Response to this questionnaire was 84 %.<br><br>**Data Analysis**: 5-point Likert scales, t-tests or chi-square tests, multilevel analysis. Analyses were performed using SPSS version 18.0 and SAS version 9.2. A p-value of <0.05 was considered significant.<br><br>**Study Quality:**<br><br>Objectives and methods are appropriate. Large intervention over 2.5 years, some drop outs.<br><br>Socio-demographics not well reported and this limited study believability as reader could not judge how this study might apply to other settings/populations.<br><br>Effect sizes discussed ("To detect small differences in diabetes incidence (Cohen's conventional effect size of 0.1), with a power of 0.8, 393 individuals were needed in each arm")	**Main Themes relevant to research question**:<br><br>**<u>Barriers to attendance:</u>**<br>Attendance of intervention-group participants at group-consultations gradually decreased from 72 % to 38 %. Reasons for missing group-consultations were 'already received enough information from the GP /nurse practitioner' (24 %), 'evening doesn't suit me' (20 %), and 'lack of time' (16 %).<br><br>**<u>Attitude of providers</u>**<br>86% of the providers reported medium or **high confidence in diabetes prevention** in primary care, while 76 % had medium or high confidence in prevention by lifestyle intervention. Of all providers, 81 % reported medium or high satisfaction with individual counselling. 23% considered the chance of **success of diabetes prevention by lifestyle counselling in primary care** low or very low.<br><br>**<u>Expertise of providers</u>**<br>Nearly all participants that had received advice were **satisfied with the level of knowledge** of both GPs (94.3 % to 100 %) and nurse practitioners (97.0 to 99.7 %) on each topic discussed.<br><br>All professionals were **confident about their level of knowledge** regarding diabetes and weight- and exercise-related topics., 80 % of the providers were confident about their basic level of dietary knowledge<br><br>Half of the participants were satisfied and 40 % was moderately **satisfied with the guidance from their GP** regarding lifestyle modification.<br><br>Seventy percent of the participants were satisfied and 25 % was moderately satisfied with the guidance from their nurse practitioner.<br><br>All nurse practitioners regarded the **training course as useful** or very useful and all would find such a course desirable or very desirable for nurse practitioners if the programme would be implemented in the Netherlands.<br><br>Eighty-five percent of the GPs and all nurse practitioners regarded themselves suitable or moderately suitable for **exercise-related guidance**. Whereas all nurse practitioners found themselves suitable (63 %) or moderately suitable (37 %) to **guide dietary** change, nearly 40 % of the GPs found themselves not suitable for nutritional counselling. Another 52 % of the GPs regarded him- or herself moderately suitable.<br><br>Lack of time and specialist knowledge (amounts of nutrients in food products,

Study details	Population and setting	Methods & Study Quality	Findings
			dietary constraints, calculation of calories in the diet) were mentioned by all providers as **barriers for guiding dietary change**.<br><br><u>**Structure and intensity of the intervention**</u><br>In total, 75 % of the providers and 86 % of the participants were satisfied with the **frequency** of the individual consultations and 68 % and 92 % respectively with their **duration**.<br><br>Professionals regarded lack of time (60 %), lack of participant motivation for lifestyle change (12 %) and lack of financial reimbursement (11 %) as important **barriers for implementation** of individual lifestyle counselling in primary care.<br><br>Seventy percent of the professionals regarded the **GP-practice as the most appropriate setting** for group-consultations on lifestyle, as it is 'a familiar environment for participants that they already relate to their health'.<br><br>The nurse practitioner, either alone (35 %) or together with the GP (23 %) or a dietician / physiotherapist (21 %) was seen as the k**ey player for organizing such consultations**.<br><br>Lack of practice space (23 %), lack of participant motivation (28 %) and lack of time of professionals (18 %) were mentioned as **organisational barriers**.<br><br>Nearly 90 % of the professionals indicated **free-of-charge exercise programmes** should be part of lifestyle interventions for diabetes prevention as they 'offer structured guidance to participants and thereby stimulate motivation'. Of the participants, 54 % was favourable to such programmes and the same percentage would favour personal counselling by a dietician.

S2: Extraction Tables

Study details	Population and setting	Methods & Study Quality	Findings
**Author**: Vermunt<br><br>**Year:** 2013<br><br>**Setting / country**: Netherlands<br><br>**Aim of study:** to explore intervention content.<br><br>**Study design**: Quantitative (questionnaire)<br><br>Part of trial: APHRODITE lifestyle intervention for the prevention of type 2 diabetes in Dutch primary care. Linked to Vermunt 2012 paper on intervention delivery<br><br>**Intervention:**<br>A 2.5-year intervention was performed in 14 general practices in the Netherlands among individuals at high risk for type 2 diabetes (n = 479) and was compared to usual care (n = 446).<br><br>Intervention consisted of individual lifestyle counselling by nurse practitioners (n = 24) and GPs (n = 48) and group-consultations.<br><br>**Usual care group:**<br>During the admission interview, participants in the usual care group received oral and written information about type 2 diabetes and a healthy lifestyle. The nurse practitioner was visited only for measurements at baseline and after 6, 18 and 30 months. Apart from the admission interview participants did not have study-related encounters with GP.	**Number of participants**:<br><br>Intervention Patients: N = 479<br>Usual care: N = 446<br><br>**Mean Age:** Not reported<br>**Gender**: Not reported	**Data collection methods**:<br><br>Participant questionnaires were filled out after 18 months of intervention<br><br>Response was 92% in the intervention group and 85% in the usual care group.<br><br>**Data Analysis**: Differences between study groups were analysed with chisquare tests using SPSS 18.0. A p-value of <0.05 was considered significant. additionally, the effect of a bonferroni adjustment for multiple comparisons was investigated (p = 0.05/15 = <0.003).<br><br>**Study Quality:**<br><br>Objectives and methods were appropriate.<br><br>Demographic data poorly reported. (see above)<br><br>Effect sizes reported(see above)	**Main Themes relevant to research question**:<br><br>Both the **motivational and volitional barriers** were highly comparable between the study groups. For all objectives an important barrier for planning change was '*I already meet the standards*'. This especially applied to the dietary fibre, total fat and physical activity objectives, with reporting-rates ranging from 56 to 66% (intervention) and from 48 to 69% (usual care). Another important factor limiting participant motivation was '*I'm satisfied with my health and/or behavior*', especially regarding weight (intervention: 26%; usual care: 35%).<br><br>For the **weight loss and physical activity objectives**, continuity (maintaining a new habit on the longer term) was an often-reported bottleneck (intervention: 13% and 12%; usual care: 14% for both). For the weight loss and fat-related objectives, temptation to snack was an important volitional barrier, with reporting-rates ranging from 19% to 32% (intervention) and from 18% to 28% (usual care). Lack of time was a bottleneck for increasing physical activity (intervention: 17%; usual care: 23%). A substantial number reported 'no difficulties' when trying to achieve dietary objectives (intervention: 33%-52%; usual care: 33% to 67%).<br><br>**<u>Motivational barriers:</u>**<br>N (%) (intervention group)<br><br>Weight loss:<br>1. Weight is healthy 29 (40)<br>2. Satisfied with weight 19 (26)<br>3. Achieved my goal 5 (7)<br><br>Increase dietary fibre intake<br>1. Eat enough dietary fibre 59 (60)<br>2. Satisfied with what I eat 10 (10)<br>3. Already took dietary fibre into account in diet 5 (5)<br><br>Reduce fat intake<br>1. Diet does not contain too much fat 44 (56)<br>2. Already took fat intake into account in diet 12 (15)<br>3. Satisfied with what I eat 4 (5)<br><br>Reduce saturated fat intake<br>1. Diet does not contain too much saturated fat 23 (37)<br>2. Already took saturated fat into account in diet 14 (23)<br>3. Satisfied with what I eat 6 (10)

Study details	Population and setting	Methods & Study Quality	Findings
**Funding**: The Netherlands Organization for Health Research and Development			Increase physical exercise<br>1. Have enough exercise 55 (66)<br>2. Physical inabilities 16 (19)<br>3. Not enough time 3 (4)<br><br>**Volitional barriers ‡**<br><br>**N (%) (intervention group)**<br><br>Weight loss<br>1. Temptation to snack 51 (26)<br>2. Continuity, relapse ** 26 (13)<br>3. Special occasions 21 (11)<br><br>Increase dietary fibre intake<br>1. No difficulties 84 (52)<br>2. Taste of products 23 (14)<br>3. Product knowledge 11 (7)<br><br>Reduce fat intake<br>1. Temptation to snack 69 (32)<br>2. No difficulties 64 (29)<br>3. Taste of products 34 (16)<br><br>Reduce saturated fat intake<br>1. No difficulties 75 (33)<br>2. Temptation to snack 44 (19)<br>3. Taste of products 31 (14)<br><br>Increase physical exercise<br>1. No difficulties 45 (22)<br>2. Not enough time 35 (17)<br>3. Continuity, relapse 26 (12)<br><br>‡ Motivational barriers were collected from non-planners; volitional barriers from initiators and achievers.

S2: Extraction Tables

Study details	Population and setting	Methods & Study Quality	Findings
**Author**: Whitford et al.<br><br>**Year**: 2002<br><br>**Setting / country**: England<br><br>**Aim of study:** to explore the attitudes and beliefs held by general practitioners (GPs) and practice nurses towards screening for Type 2 diabetes.<br><br>**Study design**: Qualitative (interviews)<br><br>**Funding**: Scientific Foundation Board of the Royal College of General Practitioners	**Number of participants**:<br><br>10 GPs and 9 practice nurses in 8 general practices<br><br>**Mean Age**: ***NR***<br><br>**Gender**: **NR**	**Data collection methods**:<br><br>Purposive sampling, based on a local diabetes survey.<br><br>Semi-structured interviews with 10 GPs and 9 practice nurses in 8 general practices in North-east England.<br><br>**Data Analysis**: Data collection and analysis proceeded in an iterative manner in accordance with grounded theory.<br><br>**Follow up**: NA<br><br>**Study Quality:**<br>Objectives were appropriate.<br><br>The sample was small (n=19) with no discussion of saturation and made it difficult to assess if sample size was adequate.<br><br>No demographic data presented so difficult to ascertain how this population could be applicable to other populations.<br><br>Appropriate methods to explore GP views but more context required.	**Main Themes relevant to research question**:<br><br>**1. Effectiveness of lifestyle interventions**<br>All the practitioners believed that Type 2 diabetes is modifiable, but their perception was that very little of this control came through patients. They expressed a belief that **patients are not able to make sufficient lifestyle changes** to impact the disease process, particularly when they are unlikely to be motivated by symptoms, the development of complications or the administration of medication or insulin.<br><br>"It's a complicated condition you know that often requires people to make lifestyle changes which are very difficult for a lot of people to make, however hard you try you know. Getting fat old ladies who live on their own, to change their diet and take more exercise and so on isn't very easy. (GP4)<br><br>**2. Resources**<br>Relief was expressed that effective drugs were available, as the skills to **promote lifestyle changes were absent or underused**.<br><br>This was compounded by a **lack of time**, as health promotion and lifestyle interventions were seen as more **time consuming** than prescribing medication.<br><br>**Workload** was a major issue, not so much in screening and diagnosis, but more in the continued **follow-up of detected cases**.<br><br>"I think there are implications of the fact we'd be doing extra screening to start with. So that's fine. and that if we do find diabetics then we're going to have to manage them, so I think what would be the implication is time and that could well be a barrier". (PN3)<br><br>**3. Attitudinal barriers to screening**<br>There was a **reluctance** to screen and promote lifestyle changes in elderly patients, who were seen as more resistant to change.<br><br>"What's the point of screening for something in an elderly person?" (GP8)

S2: Extraction Tables

Study details	Population and setting	Methods	Findings
**Author**: Whittemore et al.<br><br>**Year**: 2009 and 2010<br><br>(1 study reported in 2 papers)<br><br>**Setting / country**: USA (New England)<br><br>**Aim of study:** To examine the reach, implementation, and a lifestyle program implemented in primary care by nurse practitioners (NP) for adults at risk for type 2 diabetes.<br><br>**Study design**: mixed-method clinical trial design and evaluation (qualitative and quantitative)<br><br>**Funding**: National institute of Health	**Number of participants:**<br><br>4 Primary Care sites recruited 58 adults at risk of diabetes from their practices (31 for the treatment group, and 27 control participants)<br><br>**Mean Age**: 48 years (intervention 43 years (control)<br><br>**Gender:** primarily female (92%)<br><br>**Other**: 45% White; 34% Black; 21% Hispanic<br><br>**Sample** was obese, moderately low-income adults at risk for type 2 diabetes.	**Data collection methods**:<br><br>A convenience sample of 4 Nurse Practitioner (NP) primary care practice sites were recruited from a regional practice-based research network for NPs in New England through a mailed invitation (22% response rate).<br><br>NPs were interviewed prior to program, at 3 months, and at completion, and a questionnaire on lifestyle counselling at baseline.<br><br>The NP sites (n = 4) were randomized to an enhanced standard care program (1 NP session and 1 nutrition session) or a lifestyle program (enhanced standard care and 6 NP sessions).<br><br>NPs recruited adults at-risk for diabetes from their practice (n = 58) with an acceptance rate of 70%.<br><br>The average length of the program was 9.3 months<br><br>**The lifestyle change program** (intervention) - based on the DPP (Diabetes Prevention Program). The goals for this program were identical to enhanced standard care, yet the approach was more intensive.<br><br>The lifestyle change program for this study provided: (a) culturally relevant education on nutrition, exercise, and T2D prevention; (b) behavioral support in collaboratively identifying lifestyle change goals and problem-solving barriers to change; and (c) motivational interviewing when participants were unable to achieve lifestyle goals. These components were identical to those utilized in the DPP.<br><br>Training for NPs at the sites randomized to the lifestyle program consisted of training on the enhanced standard care protocol, self-study	**Main Themes relevant to research question:**<br><br>*(key NP= Nurse practitioner; T2D= Type 2 diabetes)*<br><br>**Attendance**<br>Participant attendance for in-person sessions was high at 96% across all sessions and all participants. Reminder phone calls for appointments and flexible rescheduling enhanced attendance. (2010)<br><br>NP phone call completion for the lifestyle program was only 37% as a result of scheduling difficulties (both providers and participants). NPs found it hard to reach people over the phone. 37% as a result of scheduling difficulties (2010)<br><br>**Implementation**<br><br>NPs reported that they felt well prepared and moderately effective in providing lifestyle counselling to adults at risk for T2D in their practice settings. (2010)<br><br>However, none of the NPs had a structured approach to T2D prevention in their practice. NPs reported that lifestyle counselling often occurred informally within the context of primary care appointments. (2010)<br><br>The NPs of the lifestyle program reported confidence in the ability to implement the educational and behavioural strategies of goal setting and problem-solving. (2009)<br><br>All NPs reported that motivational interviewing was the most challenging aspect of the protocol to implement. The NPs reported difficulty in building motivation to change and in helping participants see that their behaviour was inconsistent with personal values and goals; however, they worked consistently at improving their skills for the duration of the study. The NPs requested additional training and expert consultation throughout the course of the study. (2009)<br><br>One factor that contributed to difficulty implementing the protocol was time as NPs were encouraged to complete sessions in 20 minutes to maintain their office schedule. (2009)<br><br>The NPs reported that study participants often discussed psychosocial issues within the context of lifestyle change (e.g., stress of job), and this sometimes precluded the ability to complete all aspects of the protocol. In this situation, participants were

S2: Extraction Tables

Study details	Population and setting	Methods	Findings
		(reading and a 45-minute DVD on motivational interviewing), two 2-hour workshops on motivational interviewing (before study, at 3 months), a 2-hour education session reviewing the lifestyle program protocols, and monthly meetings with the primary investigator. Consultation with an expert on motivational interviewing was available throughout the study. Study nutritionists provided nutrition sessions at all sites.<br><br>**Enhanced standard care** (control)—<br>All participants (regardless of group assignment) received written information about diabetes prevention, a 20- to 30-minute individual session with their NP on the importance of a healthy lifestyle for the prevention of T2D, and a 45-minute individual session with a nutritionist hired for the study.<br><br>Participants were encouraged to follow a healthy diet (limit calories, fat, and processed foods); to lose 5-7% of their initial weight through diet and exercise; and to increase their exercise gradually with a goal of at least 30 minutes of exercise (e.g., walking) 5 days per week.<br><br>Training for NPs at the sites randomized to enhanced standard care only and study nutritionists consisted of a 2- hour education session reviewing the study protocols. Monthly meetings were conducted to discuss any implementation questions.<br><br>**Data Analysis**:<br>Reach and implementation were analysed with descriptive statistics and content analysis of NP interviews and process notes.<br><br>**Follow up**: Attendance was high (98%) and attrition low (12%).	encouraged to complete the session content at home using the standardized education hand-outs. (2009)<br><br>NPs of the lifestyle group reported that the program implementation was easier over time and that they were able to individualize the session content to best meet the needs of a participant. Patients were able to focus on the content that mattered to them, and any material not covered was suggested as extra reading for patients in a hand-out. (2010)<br><br>While there were some notable issues in the implementation of study protocols in this study (i.e., difficulty completing phone sessions, longer duration of program, frequent rescheduling of participant appointments), protocol implementation of in-person sessions was very good. (2009)<br><br>**Training**<br>The NPs were able to implement successfully a lifestyle program aimed at T2D prevention within the context of primary care (i.e.,20-minute sessions) and without considerable training. (2009)<br><br>**Patient satisfaction**<br>Participants of the lifestyle program were more satisfied with the program ($p = .05$) when compared to standard care. (2010)<br><br>Intervention participants who were interviewed at the end of program ($n = 26$) reported that they enjoyed the study and felt that it was a good experience. (2010)<br><br>They felt that the number and length of sessions were adequate and they appreciated the flexibility in scheduling appointments. (2010)<br><br>Participants of the control group reported that the number of appointments was not enough and that they needed more sessions and follow-up on progress. For example, one participant stated that her motivation declined after she realized no one was tracking her.<br><br>**Participant Motivation**<br>Some participants were concerned about being at risk for T2D, not wanting to have to take medicine, and wanting to know more about health. Others wanted to lose weight, were motivated by having to be weighed on a regular basis, and felt that having to be accountable to someone for their behavior was important. (2010)<br><br>Formal meetings and feeling like they completed the program were also reported as motivating to some participants. (2010)

S2: Extraction Tables

Study details	Population and setting	Methods	Findings
		**Study Quality:**<br>Both papers assessed as one.<br><br>Objectives, methods, and data collection and analysis were appropriate.<br><br>Medium sized sample and "sample size determined by a power analysis, recruiting approximately 20% of what would be necessary for a full test of the hypothesis"	Participants of both groups commented very positively on having the program provided in a familiar place by a familiar provider. As one female participant stated, "Knowing my NP made me more comfortable . . . the rapport that we have was helpful . . . and it motivated me to come to sessions." (2010)<br><br>**What patients learned in lifestyle intervention**<br>The most common responses was that participants learned about portion sizes, food labels, better food choices, the relationship of weight loss to prevention of diabetes, and the importance of exercise.<br><br>Several participants of the lifestyle group reported that they learned about themselves. "The program made me more aware of my bad habits." "I learned about the excuses I make not to exercise." (2010)<br><br>Others learned how to encourage themselves to do things they do not enjoy but that are healthy, such as exercise. One participant learned "to continue even after messing up." (2010)<br><br>**Behaviour changes**<br>positive dietary changes, such as increasing vegetables, decreasing portions, cold cuts, sugar, and soda, buying less junk food, reading labels, and eliminating snacking at night. (2010)<br><br>Participants also reported exercising, making exercise a priority, and increasing physical activity during the day. (2010)<br><br>**Barriers to dietary change**<br><br>included stress, time, and financial resources. Some participants reported that eating a healthy diet was expensive and time-consuming. (2010)<br><br>Others had the greatest difficulty with limiting favourite cultural foods (i.e., rice and beans), portion control, or social engagements. Barriers to exercise included the weather and other physical conditions that caused pain or limited mobility. (2010)<br><br>**Suggestions for improvement**<br>More sessions and content by control group participants, greater support for exercise, and more sessions with the nutritionist. (2010)<br><br>Many participants recognized the challenges of persisting with lifestyle change and the need for on-going professional support. (2010)

S2: Extraction Tables

Study details	Population and setting	Methods & Study Quality	Findings
**Author**: Williams et al.<br><br>**Year**: 2004<br><br>**Setting / country**: Wales<br><br>**Aim of study:** To explore the views of general practitioners and practice nurses about the detection and management of people at risk of developing type 2 diabetes.<br><br>**Study design**: Qualitative (focus groups)<br><br>**Funding**: GlaxoSmithKline (paid hospitality costs and the practice honoraria)	**Number of participants**:<br><br>General practitioners (n=21) and practice nurses (n=22) from 21 practices<br><br>**Mean Age**: NR<br><br>**Gender**: NR	**Data collection methods**:<br><br>All 41 practices in one local health board area in Wales were invited to participate in one of three focus groups<br><br>21 (51% response rate) practices were represented at the sessions.<br><br>General practitioners and practice nurses participated in multi-professional focus groups (3 hours long).<br><br>**Data Analysis**: Opinions of participants were analysed into themes and sub-themes according to focus group content analysis methodology to search for 'markers of text'.<br><br>**Follow up**: N/A<br><br>**Study Quality:**<br><br>Objectives, methods, and data collection and analysis were appropriate.<br><br>Demographics were poorly reported so it was difficult to judge study applicability.<br><br>Use of interviews was justified and findings adequately discussed.	**Main Themes relevant to research question**:<br><br>A few practices have identified pragmatic means to begin to identify and follow up these patients. To deliver the promise of the primary prevention of type 2 diabetes, steps need to be taken to enable primary care to do this, or else radical alternative strategies involving other components of the National Health Service and/or the participation of other agencies need to be developed.<br><br>**1. 'Detection of those at high risk' and 'primary care activities'.**<br><br>There are '**pessimistic'** views of primary care activities in relation to the detection of those at risk and the more '**optimistic'** view 'pessimistic' and 'optimistic' are our own shorthand terms and were not used by the participants). Part of the 'pessimism' was related to the existing **workload** in managing those who already have diabetes:<br><br>'I have been involved in a diabetic clinic which has been less than 3 years. [Numbers] have gone up from 170 to 245 diabetics, you know we are really sort of stretched in handling them.' (Focus group 2, GP 3.)<br><br>Considerable **frustration** and some anger was expressed about **primary care not** being the **appropriate setting for** the detection and management of those at risk of developing type 2 diabetes — primary care being regarded as a 'treatment' and not a 'screening' service:<br><br>'The biggest thing in this is ... we are being expected to be a screening service which we have never been funded to be, we are a treatment service and all of a sudden society is expecting us to put virtually everyone who comes through the door of the surgery through some kind of scanning mechanism for every single part of their body and you happen to be looking at diabetes and coronary artery disease, which is a separately funded subject, but we have got all the rest of it as well, all the renal stuff, the orthopaedics, the eye stuff and the GI [gastrointestinal] stuff … ' (Focus group 1, GP 9.)<br><br>The required **resources were not available** (and never would be) for these additional activities:<br><br>'I just don't think we have got the resources to add these precursors to the already known diabetics.' (Focus

Study details	Population and setting	Methods & Study Quality	Findings
			group 2, GP 19.)<br><br>'We will never have the resources, it's total pie in the sky if you will, then you're down to prioritisation level, you can go in to our waiting room and, what, 33% of the British population's fat. It's about 55% in [local place]!' (Focus group 1, GP 9.)<br><br>There was also concern that primary care would be **abandoning its generalist role**, with increasing emphasis on **specialisation**. There was clear tension between wishing to remain generalists and the **pressure to become primary care specialists in topics such as diabetes**:<br><br>'We [nurses in primary care] are going to become specialised in chronic disease management. You're going to have your respiratory care nurses, your diabetic nurses and CHD [coronary heart disease] nurses … I think the role of the practice nurse is limited I think that it is eventually going to be phased out.' (Focus group 1, nurse 16.)<br><br>The more '**optimistic' view** (held by a minority of those who attended the sessions) was that this was a **problem that primary care should tackle**, although the issue of **resource** should not be ignored:<br><br>'Yes, this is an important group, the impaired fasting glycaemias, and so we need to put more resources into those really to screen them and pick them up more quickly but as you say it is a question of finances and resources to do that really.' (Focus group 3, nurse 6.)<br><br>An additional point in favour was that people at high risk of type 2 diabetes were often **identified as a result of their comorbidity**, particularly in relation to their cardiovascular risk, and that they should be followed up:<br><br>'You should be seeing them anyhow early because they are hypertensive or become hypertensive and that is how they get picked up as being impaired glucose tolerance, so they are already a population that we are probably seeing.' (Focus group 3, GP 3.)<br><br>'A lot of them are being seen … as you say in the other clinics, in the hypertensive clinic, coronary heart disease

S2: Extraction Tables

Study details	Population and setting	Methods & Study Quality	Findings
			…' (Focus group 3, nurse 8.)<br><br>Incorporating patients into the **existing diabetes clinic was a pragmatic way to following them up**. However, this 'optimistic' attitude was coupled with a concern that there were **insufficient resources available** to accomplish these activities and that, once identified, these individuals would 'invariably' develop diabetes.<br><br>'Once they are picked up we then incorporate them into our diabetic clinic and [they] get seen routinely, then as follow-up after that, and invariably they then become diabetic in due course.' (Focus group 3, nurse 6.)<br><br>**2. 'Patient factors'**<br><br>There was a **sympathetic but cynical attitude** to the perception of **low motivation** of **patients** to **modify lifestyle** behaviour to reduce risk, especially in relation to weight loss:<br><br>'I mean you are talking about asking them [patients] to change their dietary habits and everyone who has ever had a weight problem knows how incredibly difficult that is, they need to be very, very motivated to do it or that's not going to happen so if they haven't got motivation we might as well save our breath really, but how you motivate them … you can try but I don't know.' (Focus group 1, GP 9.)<br><br>**Creating motivation** in patients is likely to be **time consuming**:<br><br>'Motivation is a big factor, I find that with my patients and sometimes if they want to lose weight they want to come to me every week to be weighed and that is very time consuming.' (Focus group 1, nurse 12.)<br><br>**Motivating asymptomatic** patients is particularly **difficult**:<br><br>'It is very difficult to motivate asymptomatic patients, someone that is symptomatic, you get them on board and [can] be very successful in the management.' (Focus group 2, GP 3.)<br><br>It was also observed that, although intense interventions in randomised controlled trials had been **shown to be effective**, there was **no evidence** to support this in

Study details	Population and setting	Methods & Study Quality	Findings
			relation to interventions **delivered through primary care**:<br><br>'The evidence for actually preventing these people going on to develop diabetes involves very intensive, expensive lifestyle intervention regimes, so the evidence that the little bit that we do is actually making an impact probably is not there.' (Focus Group 2, Doctor 7.)<br><br>**3. 'Responsibility for prevention'.**<br><br>Given the above, it is unsurprising that the majority opinion was that impaired glucose tolerance and impaired fasting glycaemia **should not be 'medicalised'** and that they were **social**, rather than medical problems:<br><br>'It [asking primary care to take on the identification and management of patients with impaired glucose tolerance and impaired fasting glycaemia] almost medicalises something which actually is a social problem.' (Focus group 1, nurse 10.)<br><br>'These people [those with impaired glucose tolerance and impaired fasting glycaemia] are not ill. Should we make them ill?' (Focus group 1, GP 15.)<br><br>The primary prevention of type 2 diabetes was the **responsibility of individuals and agencies other than primary care** — indeed, of agencies outside the health sector:<br><br>'I also think they [the patients] have responsibility, maybe it is the government's responsibility.' (Focus group 2, nurse 6.) 'These issues should be dealt with through education and political pressure.' (Focus group 2, GP 21.) 'Schools. Education in schools is a huge, huge part of it.' (Focus group 1, GP 9.)<br><br>'What used to happen in schools — you used to get the school meals ... [with] veg and something else and some fruit and they had playing fields — they used to go running around and do gym and PE and kids were safe to walk to school. What has happened [is] they've sold off all the ... playing fields, give them a ... lunch. It's all chips and pizza and this. They are not safe to walk to school because there are enough idiots around trying to do things to them and they [children] are getting fatter and fatter and fatter.' (Focus group 1, GP 15)

S2: Extraction Tables

Study details	Population and setting	Methods	Findings
**Author**: Wylie<br><br>**Year:** 2002<br><br>**Setting / country**: UK, North East England<br><br>**Aim of study:** To investigate general practitioners' knowledge of and attitudes to impaired glucose tolerance.<br><br>**Study design**: qualitative and quantitative study with semi-structured interviews focus groups, and questionnaires.<br><br>**Funding**: Northern and Yorkshire Regional Health Authority	**Number of participants**:<br><br>34 general practitioners in five primary care groups<br><br>**Mean Age**: 44 years (focus groups) 41 years (interviews)<br><br>**Gender**:<br>18 men, 8 women (focus groups)<br>6 men, 2 women (interviews)<br><br>**Other**:<br><br>Average of 11 (range 1-27) years' experience in general practice. (Focus groups)<br><br>Average of 12 (range 4-24) years' experience in general practice (interviews)<br><br>The 30 general practitioners who either declined or failed to attend were similar in terms of sex and practice characteristics (Focus groups).<br><br>All general practitioners invited agreed to take part in interviews.	**Data collection methods**:<br><br>**Focus groups**<br><br>Stratified random from general practitioners from lists supplied primary care groups. Contact by telephone followed by invitation letter.<br><br>26 general practitioners participated (group was split into four separate focus groups) with an average duration of 75 minutes.<br><br>Before each focus group, participants completed a questionnaire designed to evaluate their knowledge of the clinical significance and prevalence of impaired glucose tolerance. Their responses were then explored in the focus group discussion. They were also given a short presentation which covers topics in diabetes.<br><br>**Semi-structured interviews**<br><br>8 participants were chosen from a list of all general practitioners in one health authority. Participants were chosen to reflect diversity in terms of age, sex, practice characteristics, and involvement in diabetes. Average duration of 35 minutes.<br><br>A questionnaire was completed verbally by all interviewees and it was followed by open ended questions concerning knowledge of the clinical significance and prevalence of impaired glucose tolerance. They were also given a short presentation which covers topics in diabetes.<br><br>**Data Analysis**: Collection and analysis of data for quantitative survey and qualitative analysis for interviews and focus groups.<br><br>Used a "pragmatic variant" grounded theory	**Main Themes relevant to research question**:<br><br>**Results from Questionnaire**<br><br>All participants were **aware of impaired glucose tolerance** as a clinical entity. However, 16 (47%) participants were **unaware of the risk** of impaired glucose tolerance progressing to type 2 diabetes, and 21 (62%) were unaware of the increased **risk of cardiovascular disease**.<br><br>17 (50%) participants had **no idea how many patients** with impaired glucose tolerance might be known to their practice, and 13 (38%) **estimated prevalence** at less than 1% .<br><br>**Focus groups and interviews**<br><br>*Three main themes emerged from data collected before participants received a presentation about impaired glucose tolerance :*<br><br>1. Low awareness of the prevalence and clinical significance of impaired glucose tolerance<br>2. Uncertainty about managing patients with impaired glucose tolerance<br>3. Support for a guideline for managing impaired glucose tolerance<br><br>*Eight main themes emerged after the presentation:*<br><br>**1. Fear of being overwhelmed by the workload involved in screening and managing patients with impaired glucose tolerance**<br>"I think we all probably fight shy of diagnosing too many people with impaired glucose tolerance, I mean, I'm sure we all do it. I mean, I occasionally get people who've had a borderline high sugar and it gets passed to the nurse for dietary intervention . . . they don't all have a glucose tolerance test; the reason for that is it involves a whole lot of workload" (FG3c)<br><br>**2. Concern that widespread screening and management of patients with impaired glucose tolerance would be impossible without extra resources**<br>"The practices simply can't be taking all the load. I think there are huge resource implications for the practices involved. Certainly there is a huge disincentive at the moment for me to find any more patients because I can't afford to treat them" (FG1a)<br><br>"It would be very difficult with the present staffing . . . I think it would be very

S2: Extraction Tables

Study details	Population and setting	Methods	Findings
		approach to analyse the data by generating categories and themes. Investigators coded the data independently to increase the reliability of the study. An iterative approach to data analysis, with analysis beginning after the first focus groups and interviews, to allow emerging themes to be explored in subsequent interviews.<br><br>**Follow up**: not applicable<br><br>**Study quality:**<br><br>Objectives, methods, and data collection and analysis were appropriate.<br><br>Mixed methods which was appropriate in addressing objectives, however possible selection bias.	difficult. We would have to have additional resources to do it" (INT2)<br><br>**3. Concern at diverting finite resources from other clinical areas**<br>"Fine, yes, in theory [we could screen for impaired glucose tolerance], but we haven't only even got diabetes to look after . . . but you've got so many things to look after and outside issues as well, so where does it stop?" (FG1e)<br><br>**4. Pessimism regarding the effectiveness of lifestyle intervention**<br>". . . we have diabetics who . . . who just totally ignore the advice you give them, and I think going further back than that and giving them advice when they haven't got diabetes as such is going to be very difficult" (INT4)<br><br>**5. Positive attitudes towards pharmacological intervention in patients with impaired glucose tolerance**<br>"Well, even that [lifestyle intervention] is a tall order for a lot of them. I just feel as though, if you're going to do this, you've really got to put them on metformin." (FG2c)<br><br>**6. Uncertainty regarding the role of general practitioners in detecting and treating impaired glucose tolerance**<br>"But that's not my job, you know; I'm a GP and I'm actually there probably not to do a lot of prevention but to actually do a little bit of tinkering with the people already ill" (FG4f)<br><br>**7. Concern that screening and treating impaired glucose tolerance is essentially medicalizing a social problem**<br><br>"I think it's on a bigger scale than us having to prevent it [type 2 diabetes] right at the end of the line. It's like us preventing suicides when there's unemployment and stress" (FG3e)<br><br>**8. Positive attitudes towards a health educational approach**<br><br>"I think health promotion must have a huge responsibility—they have a huge budget . . . I mean, hundreds of thousands of pounds go into health promotion. Why can't they organise themselves and, if needs be, set up opportunistic screening at supermarkets .
